# Recent advances in nanoparticle applications in respiratory disorders: a review

**DOI:** 10.3389/fphar.2023.1059343

**Published:** 2023-07-19

**Authors:** Mohammad Ehsan Taghavizadeh Yazdi, Mohsen Qayoomian, Sima Beigoli, Mohammad Hossein Boskabady

**Affiliations:** ^1^ Applied Biomedical Research Center, Mashhad University of Medical Sciences, Mashhad, Iran; ^2^ Mashhad University of Medical Sciences, Mashhad, Razavi Khorasan, Iran; ^3^ Department of Physiology, School of Medicine, Mashhad University of Medical Sciences, Mashhad, Iran

**Keywords:** nanomedicine, nanoparticle, respiratory disorders, drug delivery, treatment

## Abstract

Various nanoparticles are used in the discovery of new nanomedicine to overcome the shortages of conventional drugs. Therefore, this article presents a comprehensive and up-to-date review of the effects of nanoparticle-based drugs in the treatment of respiratory disorders, including both basic and clinical studies. Databases, including PubMed, Web of Knowledge, and Scopus, were searched until the end of August 2022 regarding the effect of nanoparticles on respiratory diseases. As a new tool, nanomedicine offered promising applications for the treatment of pulmonary diseases. The basic composition and intrinsic characteristics of nanomaterials showed their effectiveness in treating pulmonary diseases. The efficiency of different nanomedicines has been demonstrated in experimental animal models of asthma, chronic obstructive pulmonary disease (COPD), pulmonary fibrosis (PF), lung cancer, lung infection, and other lung disorders, confirming their function in the improvement of respiratory disorders. Various types of nanomaterials, such as carbon nanotubes, dendrimers, polymeric nanomaterials, liposomes, quantum dots, and metal and metal oxide nanoparticles, have demonstrated therapeutic effects on respiratory disorders, which may lead to new possible remedies for various respiratory illnesses that could increase drug efficacy and decrease side effects.

## 1 Introduction

According to the World Health Organization (WHO), respiratory diseases caused the deaths of at least 9 million people worldwide and resulted in numerous disabilities in 2016. These diseases accounted for approximately 15% of all deaths (Horváth et al., 2017). Lower respiratory infections, pneumonia, bronchitis, tuberculosis, pharyngitis, laryngitis, and the common cold, as well as obstructive lung disorders, such as asthma, chronic obstructive pulmonary disease (COPD), and lung cancer, are among the most prevalent respiratory diseases. The main causes of respiratory diseases are air pollution, genetic attributes, and infections caused by viruses and bacteria. The lower respiratory airways could easily be infected by airborne diseases and infections, influencing the lower airways and leading to acute respiratory infections (Thorlund et al., 2020). The recently known acute infection caused by the new coronavirus (COVID-19) and the older ones by severe acute respiratory syndrome coronavirus (SARS-CoV), Middle East respiratory syndrome coronavirus (MERS-CoV), and avian influenza A (H7N9) can cause severe respiratory infection. Chronic respiratory diseases are caused by genetic disorders and air pollution, including cystic fibrosis, asthma, COPD, hay fever, and lung cancer. The treatment priority goal is to improve patients’ quality of life by repairing or restoring the functions of the respiratory system (Thorlund et al., 2020). The main goals in the treatment of respiratory disorders are to provide a suitable treatment for ameliorating disease symptoms, reducing the casualties and mortality rate, increasing the quality of life, and decreasing the side effects of drugs.

The need for innovative treatment methods that can overcome obstacles, including drug resistance, low drug efficiency, side effects, and costs, may justify the use of nano-based drugs. In recent years, new doors have been opened to treat respiratory disorders with the advent of nanomedicine and nanotechnology. Nanoparticles (NPs) have a very high surface-to-volume ratio because of their nano-scale dimensions (Yazdi et al., 2021). This feature allows multiple ligands to be attached to the surface and increases the tendency to develop multiple covalent bonds and simultaneously use in targeted therapy by an appropriate design (Ghorani-Azam et al., 2022). Other characteristics of nanoparticles are increased chemico-biological stability, the possibility of binding to both hydrophilic and hydrophobic drugs, and the capability to be prescribed via inhalation or injection (Deng et al., 2021; Luo et al., 2021), which depends entirely on the physicochemical characteristics of the nano-carrier and the chemical structure of the reagents. Aerosol drug delivery is one of the popular drug delivery systems that can be used for respiratory and non-respiratory disorders (Bianco et al., 2021; Vartak et al., 2021). The size of nanoparticles would determine the deposition spot in the respiratory tract. For example, 1 nm nanoparticles accumulated over lower airways, but they usually come out from the respiratory system with expired air; 5 nm particles were placed in the tracheal and bronchi; and 20 nm particles were localized in the upper airway regions (Shakerimanesh et al., 2022).

**FIGURE 1:**
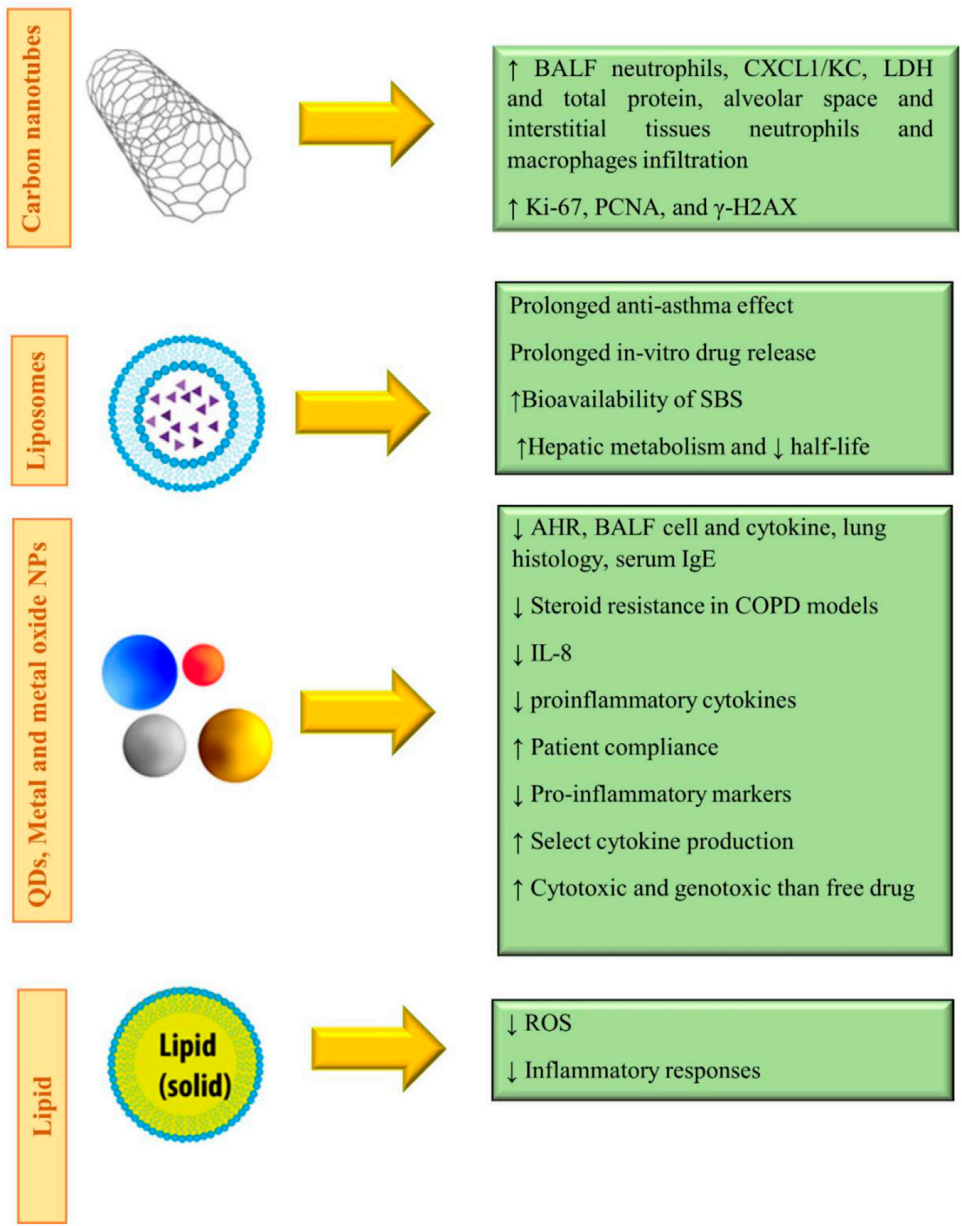
General applications of NPs for possible treatment of various disorders and their possible mechanisms of action.

The nano-systems could have higher efficacy than conventional drugs due to functionalized for targeted delivery, increased biocompatibility, and reduced adverse effects (Mousavi-Kouhi et al., 2021a). Different types of nanoparticles, including organic and inorganic nanoparticles, were available for biological application, which should be explained more thoroughly for designing new nano-systems for specific purposes. In this review article, the NPs that were used in the treatment of respiratory disorders were presented to form new ideas for more convenient future medications. Figure 1 shows the general applications of NPs for the possible treatment of various disorders and their possible mechanisms of action.

## 2 Materials and methods

*In vitro* and *i*n *vivo* studies regarding the influence of NPs on respiratory diseases were searched using online databases, including PubMed, Web of Knowledge, and Scopus, from 2015 to the end of August 2022. For this purpose, keywords including (Nanomedicine), (respiratory disorders), and (respiratory, pulmonary or lung diseases), (name of respiratory diseases individually), (nanoparticles), (inorganic nanocarrier or nanoparticle), (organic nanocarrier or nanoparticle), and (name of nanoparticles individually) were used. Furthermore, the effects of the nanoparticles on lung cancers and lung infections were covered in the current review article. Only articles published in the English language and well-known international journals were included in this review manuscript.

**FIGURE 2:**
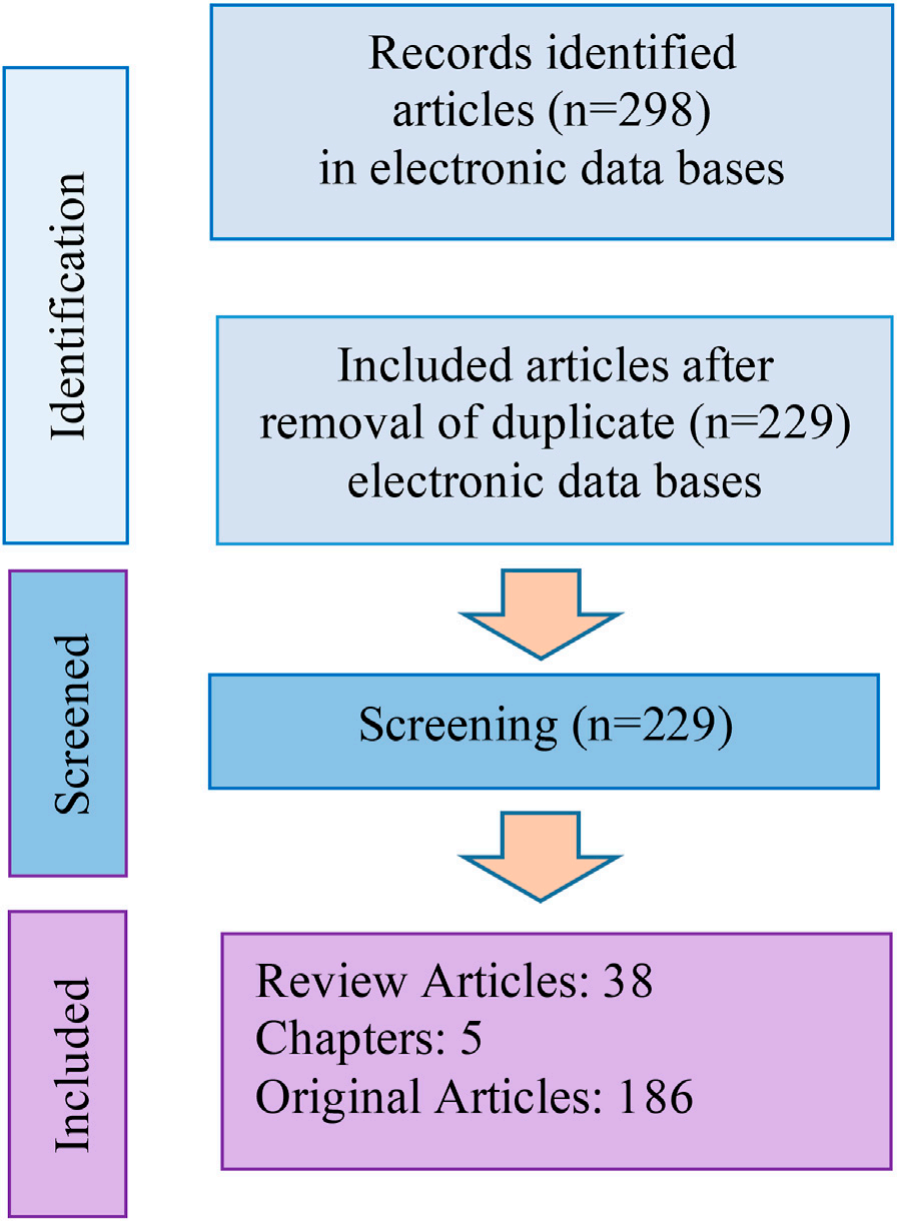
Flowchart of the process for selecting studies for this review.

Totally, 298 articles were selected from internet sources, of which 80 articles were duplicates. The study flowchart is shown in Figure 2.

## 3 Nanoparticles for biomedical applications

Nanotechnology has the potential to revolutionize the field of chemotherapy (Vahideh Mohammadzadeh et al., 2022). Nanoscale platforms aim to increase treatment efficacy and localize drug delivery, resulting in reduced dosage, fewer systemic side effects, controlling biodistribution, modulating pharmacokinetics (release kinetics and mechanism), and finally improving patient compliance (Akhter et al., 2018). Nano-architectures could be used to encapsulate different drugs, especially hydrophobic drugs. Nanoparticles can be thought of as tiny packages that contain drugs and can be delivered to specific locations in the body (Mousavi-Kouhi et al., 2021b).

The major organic-based platforms for nanotechnology include carbon nanodiamonds, nanotubes, graphene, graphene oxide, fullerenes, dendrimers, polymeric particles, solid lipid hybrid nanoparticles, liposomes (most popular), pyrosomes, nanoemulsions, and graphene/carbon quantum dots (QDs) (Ashna et al., 2020; Naciute et al., 2020). Inorganic-based drug delivery systems comprise metal and metal oxide nanoparticles (especially gold for diagnosis and magnetic nanoparticles), metallic quantum dots, and porous materials (Javanbakht et al., 2020; Parra-Nieto et al., 2020) (Figure 2).

### 3.1 Nanoparticles used for the treatment of respiratory disorders

Several types of nanoparticle drugs have been used for the treatment of different respiratory disorders, and the most important types are briefly described in the following sections.

#### 3.1.1 Carbon-based nanomaterials

Carbon-based materials play important roles in the advancement of material science, including a) traditional industrial carbon, such as activated carbon, b) new industrial carbon, such as carbon fibers, and c) new carbon nanomaterials, such as graphene and carbon nanotubes. Primary investigation and uses of carbon-based materials are general in the interdisciplinary fields, although macroscopic carbon material lacks a suitable band gap, making it problematic to act as an efficient fluorescent material (Liu et al., 2020). Carbon nanodiamonds and carbon nanotubes are light, strong, highly conductive, and versatile. The drug delivery systems of this type of nanoparticles could be functionalized to prepare robust diagnostic tools (Chauhan et al., 2020). Carbon nanotubes are inhalable but would cause health risks (Oberdörster et al., 2015), such as lung fibrosis and inflammation (Poulsen et al., 2016); hence, they are not suitable for the treatment of respiratory diseases. In recent years, by developing the substantial properties of diverse newly invented carbon-based nanomaterials, these have been modified and widely employed in biology, medicine, and pharmacology (Maiti et al., 2019).

#### 3.1.2 Dendrimers

**FIGURE 3:**
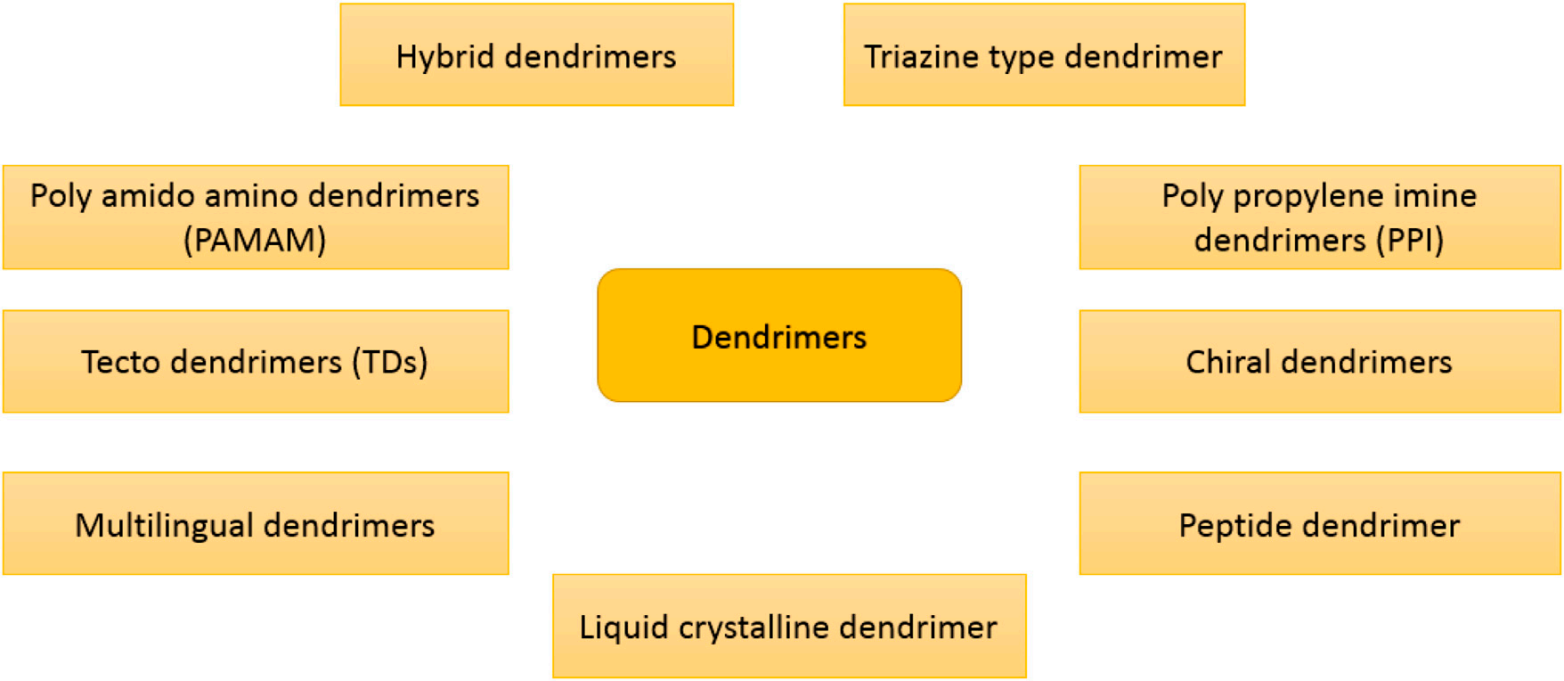
Classification of dendrimers based on their physical and chemical characteristics.

The term “dendrimer” was derived from the Greek word dendron, which means “tree” because dendrimers have a branching structure that resembles a tree (Steinman, 2007). A dendrimer consists of dendrons, which are arms initiated from the central core and can be classified on the basis of physicochemical characteristics (Ambekar et al., 2020). Dendrimers are radially extended hyperbranched architectures with high versatility that can be modified for different medical purposes such as drug delivery, gene delivery, cancer targeting, and diagnosis. By changing the functional groups, the hydrophobic zones could be changed/engineered to hold certain hydrophobic drugs (Wang et al., 2016). In this regard, poly(amidoamine) (PAMAM) is a dendrimer with amide and amine functionalities. Dendrimers can be categorized based on their physicochemical construction, as shown in Figure 3. Monodispersity is one of the exclusive characteristics of dendrimers that can be responsible for the replication of dendrimers at a scalable rank. Dendrimers are extensively used as drug delivery (Zenze et al., 2023), biosensors (Zhou et al., 2023), pharmacology (Pérez-Carrión and Posadas, 2023), antibiotics (Xu et al., 2023), and gene editing (Liu et al., 2023). Today, many types of dendrimers, such as polylysine, polyethylene glycol, and PAMAM, are widely used as nanocarriers for the treatment of diseases (Yousefi et al., 2020). Gold and iron nanoparticles, which are well-known for the treatment of cancer, such as paclitaxel, cisplatin, and doxorubicin are loaded in dendrimer (Khemtong et al., 2009; Zarharan et al., 2023). The solubility of the hydrophobic drugs could be enhanced using embedding in dendrimers (Zhang et al., 2011). Dendrimer-based nanocarriers need to be functionalized with biomolecules to overcome the cationic toxicity of dendrimers, which can be easily carried out due to the large amino groups in dendrimers (Ambekar et al., 2020).

#### 3.1.3 Polymeric nanoparticles

Polymeric nanoparticles can be defined as solid colloidal particles, and they are preferably made up of polymers attained from a natural or synthetic basis (Yazdi et al., 2020). Polymeric nanoparticles can be separated into classes of hydrogels, nanospheres, and nanocapsules, examples of the last being polymeric micelles and polymersomes. Hydrogels are polymeric materials with a significant number of attached hydrophilic groups and have a three-dimensional network that helps them absorb a large amount of water or dissolved drugs chemically or physically. Nanocapsule polymeric NPs are the ones in which the remedial element is encapsulated inside a polymeric capsule shell, whereas nanosphere polymeric NPs are the ones in which drugs or other solid particles are implanted into a polymeric matrix. Polymeric nanoparticles deliver the drug to a specific site and deliver the drug at a specific amount, which is valuable for the treatment of various diseases (Sur et al., 2019).

#### 3.1.4 Liposomes

Liposomes (LPs) are vesicles formed from concentric lipid bilayers, so-called lamellae, which are spontaneously formed when certain lipids are hydrated in aqueous media. LPs are very similar to self-assembling colloidal polymeric nanoparticles but constructed from lipid bilayers. Lipids have both hydrophilic and hydrophobic sides that can wrap into spherical or oval-type shapes (Saraf et al., 2020). Nanoparticles similar to liposomes are phytosomes, micelles, nanoemulsions, and solid lipid nanoparticles (Gorain et al., 2020). More specifically, liposomes have an aqueous core that is perfect for encapsulating hydrophilic drugs (New, 1990). In hydrophobic drugs, it could also be placed between the lipid bilayer of the cell membrane in a phagosomal form (Kumar et al., 2020). LPs used in biology and medicine display a range of benefits comprising high drug/lipid proportion loading efficacy, simplicity of synthesis in a size-controlled way, great stability, manageable delivery kinetics, and biocompatibility (Zylberberg and Matosevic, 2016). Moreover, LPs can be extra equipped with functional moieties to increase their performances in terms of increased circulation half-life, targeted delivery, and intracellular permeation capability (Kim, 2016). Consequently, LPs show better pharmacokinetics and biodistribution outlines than numerous other drug transporters in clinical research, thus improving the pharmacokinetics of encapsulated remedial factors while keeping them and preventing their associated side effects and drug reactions (El-Hammadi and Arias, 2019).

#### 3.1.5 Lipid nanocarriers

Lipids make up selectively permeable cell walls; hence, liposomes could be the most permeable and biocompatible nanocarriers in the body. Most of the FDA-approved nanoparticles are based on liposomes, but they encounter major limitations. Liposomes are prone to oxidative degradation and drug leakage when continuously exposed to aqueous solution and body fluids (Lee and Thompson, 2017). Nanoemulsions and solid lipid nanoparticles could be better candidates than liposomes, which cover a broader range of pharmaceuticals and more affordable drug delivery systems (Roy et al., 2022). Lipid-based nanocarriers could dominate the difficulties of low availability for oral treatment with poor water solubility. Lipid NPs have the features of nanosized particles and lipid solubility, leading to increased pharmacokinetic and biocompatibility and decreased harmfulness and enabling scale-up for industrialized fabrication (Dumont et al., 2018). Their digestion involves the dispersion of fat globules into an emulsion with great surface area, enzyme hydrolysis of the triglyceride lipid at the lipid–water interface, and the dispersion of the bio-product into the absorbed formula (Joyce et al., 2016). Also, the encapsulation of drugs or bio-agents using lipid-based nanocarriers can decrease the food intake effects on absorption and variation among topics because of their controlled release (Hsu et al., 2019).

#### 3.1.6 Quantum dots

**FIGURE 4:**
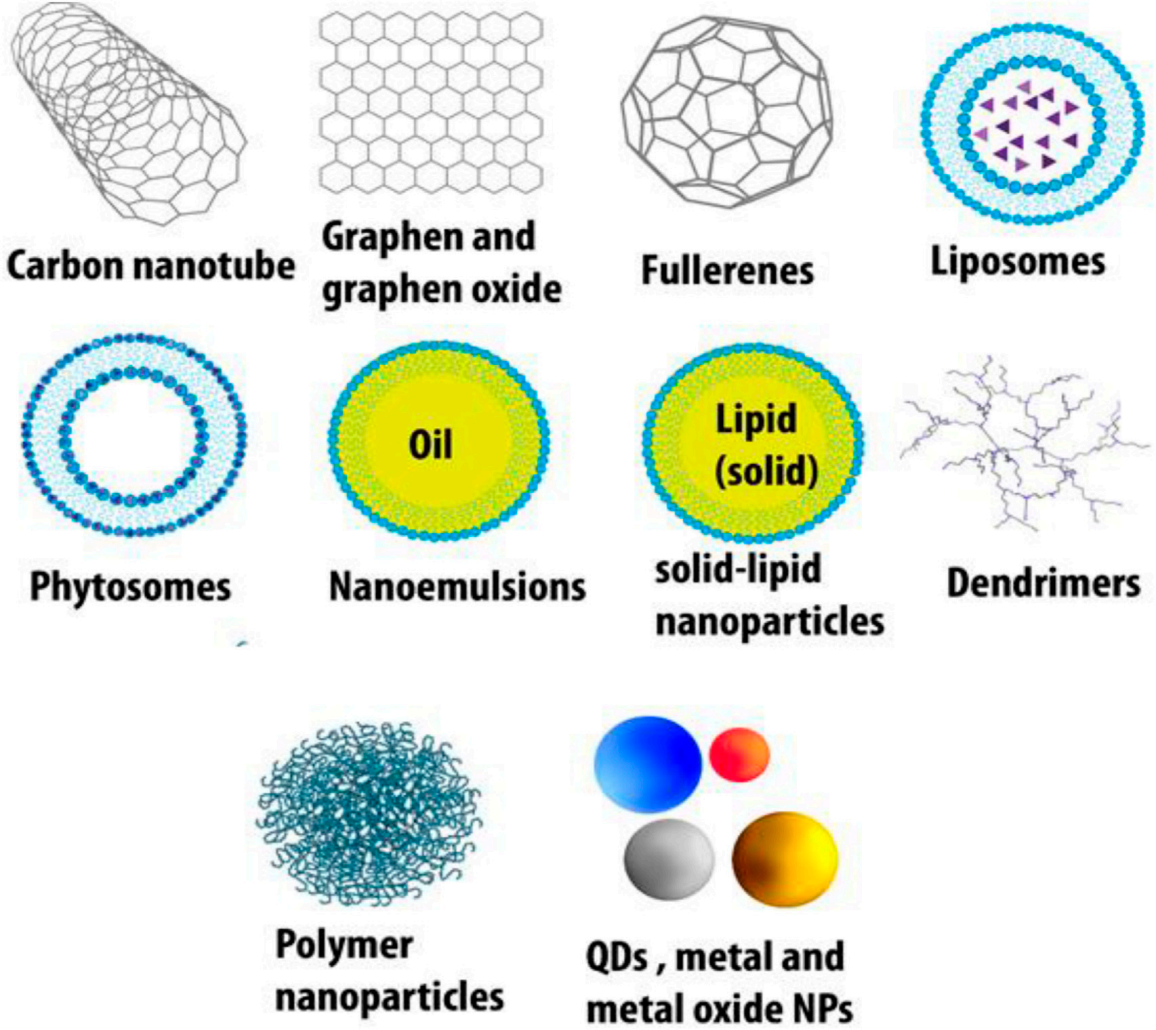
Different types of NPs applied in respiratory diseases.

Quantum dots (QDs) have been known as an innovation in nanoscience for semiconductor inorganic crystals that comprise variable numbers of electrons, which occupy well-defined and distinct quantum statuses (Reshma and Mohanan, 2019). Moreover, QDs are small-sized particles that prepare a wide range of flexible element ratios, which could cause fluorescent properties (Hu et al., 2019). The construction of QDs could be simply controlled while obeying the fundamental of quantum confinement (Bai et al., 2018). Specifically, QDs are small semiconducting particles with typical sizes ranging from 1 to 10 nm. They can make a valuable contribution in an extensive variety of applications and may substitute for many expensive materials now used for fluorescent tagging and cell imaging because of their facile synthesis, high photo-stability, high resistance to metabolic degradation, and brightness. QDs need little energy to be excited (blue light), and the emitted light from QDs has a narrow emission spectrum at longer wavelengths than the absorbed light. Moreover, the emitted wavelength can be controlled by controlling the size of the QD. QDs could be used for the synthesis of multifunctional nanoparticles that could detect cancer cells and visualize, track, kill, and monitor them. Typical QDs made from carbon, graphene, cadmium or zinc chalcogenides, phosphides, and indium arsenide are placed in both organic and inorganic categories (Ruiyi et al., 2020). Figure 4 shows the role and potential of some QDs in the respiratory system.

#### 3.1.7 Metal and metal oxide nanoparticles

Metal and metal oxide nanoparticles are inorganic-based nanocarriers that can be used as a means to transfer small drug molecules and large ones, such as DNA or proteins, throughout the body (Mobaraki et al., 2021). The surface of the nanoparticle could be coated with a protective layer for stability issues in the body fluids and be tagged by different ligands on the outside to attach preferentially to receptors of the cancer cells (Zarei et al., 2021). For example, gold nanoparticles could be PEGylated or functionalized with fluorescent labels, protein, or other targeting agents, which can penetrate the cell membranes while carrying the DNA or the drug (Song et al., 2018). Metal and metal oxide nanoparticles display altered physical and chemical activities and are different from their native bulk materials in some things, which consist of their surface, optical, thermal, and electrical properties. Metal and metal oxide NPs are synthesized using the addition of reducing or oxidizing/precipitating factors during their synthesis, respectively (Hoda Zarharan et al., 2023). Several factors are involved in NP reactivity with biomolecules, which comprise nanoparticles’ size, shape, surface activities, stability, and the process of fabrication (Rastogi et al., 2017). Figure 4 demonstrates different types of NPs applied to respiratory diseases.

### 3.2 Methods for NP establishment for lung delivery

A number of conventional methods have been described for the expansion of particulate material for specific delivery to the lung. Nevertheless, there are limitations such as shape, size, and distribution (Patil and Sarasija, 2012). The physicochemical features of any particle are the key agents defining its flow activity, which impacts the particle transport and deposition on the pulmonary structure (Tsuda et al., 2013). However, the influences of inhaled NPs are obviously far for the treatment of non-lung disorders, especially the cardiovascular structure and other highly vascularized organs, such as brain diseases (Mostovenko et al., 2022).

Inhaled powders based on various NP types, such as polymeric nanoparticles, liposomes, solid lipid nanoparticles, and mesoporous silica nanoparticles, were studied for delivery to the respiratory system but mostly for polymeric and lipid nanoparticle types of NPs (Chan et al., 2023). In addition, the clinical study for the liposome inhaler form is also a development for the delivery system (Forest and Pourchez, 2022).

#### 3.2.1 Spray drying

In this method, for thermolabile matters such as protein and insulin to be dried, they are atomized into tiny droplets and driven radially into a moving stream of hot drying medium (Pramanik et al., 2021). This method was planned to make submicron particles with greater products in the primary engineering sector, even for massive-scale formats. However, it did not get pleasure with NP preparation due to, for example, little separation capacity. Investigations using inhaled NPs dispersed in watery droplets propose that mucus clearance could be overcome by NPs, probably because of the fast movement of particles to the airway epithelium through surface energetics (Sham et al., 2004). Hence, NPs might be potential vehicles for effectual transporting drugs to the epithelium, although causing undesirable mucociliary clearance.

#### 3.2.2 Spray-freeze-drying manner

Spray-freeze-drying (SFD) is a drying manner that first found uses in the arena of pharmacological technologies and has a variety of applications in pharmacological and nutrition technology. This manner is still a distinctive way to increase insufficient water-soluble constituents (Adali et al., 2020). It is an advanced particle establishment manner in which a liquid matter is intended to spray in hot air or nitrogen, followed by lyophilization. The feed rate, which impacts the aerodynamic diameter and size, is the chief cause for decreasing the efficacy of this method (Pramanik et al., 2021). Although some studies have shown the merits and exclusive properties of SFD, the technique has some limitations. SFD has high capital and operational costs, chiefly due to low-pressure and low-temperature necessities. In addition, most developed SFD units are batch-type and unsuitable for commercial usage (Vishali et al., 2019).

#### 3.2.3 Solvent precipitation technique

This is a direct-controlled crystallization technique that involves sono-crystallization and microprecipitation to distribute the narrow drug particle size in a crystalline form (Patil and Sarasija, 2012). Sono-crystallization is an exclusive technique for planning an NP system. The energy involved in this procedure will ameliorate the target media’s nucleation level more than the others, where this manner is not incorporated (Pramanik et al., 2021).

The use of various types of inhaled NPs was indicated in different sections of this review.

## 4 The effects of nanoparticles on respiratory diseases

### 4.1 Asthma

Asthma is an inflammatory disorder of the lungs characterized by reversible airway obstruction, bronchial hyper-responsiveness, and chronic airway inflammation (Cukic et al., 2012). Long-term exposure to irritants causes an inflammatory response in the lungs, resulting in narrowing the small airways and lung tissue damage (Yang et al., 2019). Anti-inflammatory effects of nanoparticles were shown in various inflammatory disorders previously. Nanoparticles increase the therapeutic effect by facilitating the delivery of the drug to the target tissue, thereby improving the deposition of the drug in the lungs (Yang et al., 2019). In several *in vitro* and *in vivo* studies, the effects of nanoparticles on asthma were reported.

#### 4.1.1 *In vitro* studies

The poly(lactic acid) nanoparticles were utilized to co-deliver theophylline and budesonide. These two compounds were co-encapsulated in the polymeric nanoparticles through the process of double emulsion solvent diffusion. The poly(lactic acid) nanoparticles can encapsulate both hydrophilic and hydrophobic drugs and be used for co-delivery purposes, although it has a hydrophobic nature. The drug release was investigated using Franz-type diffusion cells using a 1:1 ratio of the simulated lung fluid and methanol in the airway epithelial cell line (16HBE14o-). Inhaled nanoparticles lead to a fine particle fraction of 75% and 48% for theophylline and budesonide, respectively. The results showed that budesonide and theophylline drug-loaded NPs are appropriate drug delivery methods for the treatment of asthma and COPD (Buhecha et al., 2019). In the other work, nanoliposomal salbutamol sulfate (SBS) dry powder inhalation for facilitated asthma therapy was used, and the results showed prolonged *in vitro* drug release of above 90%. In this study, researchers overcome the deprived oral bioavailability of SBS because of its hepatic metabolism and short half-life (Honmane et al., 2019). Elimination of low-solubility issues with curcumin by liposomal curcumin improved patient compliance due to the liposomal form. The suppression of proinflammatory markers, such as IL-6, IL-8, IL-1β, and TNF-α, by curcumin liposomes is the result of this study (Ng et al., 2018).

In another work, quercetin-loaded liquid crystalline NPs (LCN) and surface-modified liquid crystalline NPs (sm-LCN) were formulated, and their anti-inflammatory activity in a human primary bronchial epithelial cell line (BCi-NS1.1) stimulated with lipopolysaccharide (LPS) was investigated. Quercetin LCN and sm-LCN meaningfully reduced the fabrication of IL-1β, IL-6, and IL-8 compared to the LPS-only group. Encapsulation of quercetin into LCN and sm-LCN increased its anti-inflammatory property compared to quercetin in DMSO. Moreover, quercetin LCN and sm-LCN displayed comparable properties to fluticasone in terms of decreasing the fabrication of IL-1β and IL-6. Therefore, quercetin-LCN and sm-LCN can be possible remedial interventions for asthma as they are effective in inhibiting the fabrication of main proinflammatory cytokines related to the development of asthma (Yong et al., 2019).

Treatment with lipid nanoparticles decreased the secretion of proinflammatory cytokines, including IL-4 and IL-13, in asthmatic mice and could significantly diminish allergic asthma by hindering the overexpression of proinflammatory cytokines in the airway (Zhang M. et al., 2022). Treatment of asthmatic mice with poly(lactic-co-glycolic acid) (PLGA)-levamisole NPs reduced airway hyper-responsiveness, eosinophils in the BALF, immunoglobulin levels, and Th2-, Th9-, and Th17-derived cytokines (Fang et al., 2022).

Asthma-induced airway inflammation can be prevented by nanoparticle administration of LNA oligonucleotides. Treatment with oligonucleotide can decrease the gene expression of mmu-miR-145a-5p, which is elevated in the animal model of disease and human asthma (Ahmad, 2022). *In vitro*, the rate of oxygen consumption (OCR) in the mitochondrial respiratory function with exposure to CuONPs (25, 50, and 100 μg/ml doses) was measured. The results indicated that the 25 and 100 μg/ml doses of CuONP decreased OCR, but the 50 μg/ml dose increased OCR (Bushueva et al., 2023).

In ovalbumin (OVA)-induced asthma model and OVA-severe (lung, heart, liver, kidney, and spleen) models, AuNP-loaded macrophages migrated to the lung tissue. The results showed that macrophages loaded with AuNPs exhibited a sixfold faster migration into the lung tissue in the OVA-severe model compared to the OVA-asthma model. Macrophage phagocytic capacity and the ability to encapsulate drugs and therapeutic particles were also increased (Kang et al., 2022).

#### 4.1.2 *In vivo* studies

Aerosolized liposome formulation for the pulmonary delivery of an anti-asthmatic treatment showed a significantly more prolonged anti-asthma effect of the R-TBH liposome compared to the R-TBH (R-terbutaline hydrochloride) solution (Li Q. et al., 2018). The modulation of an asthmatic response using TiO_2_ or AuNPs in a murine model of diisocyanate-induced asthma was studied. The results displayed that a low, intrapulmonary doses of NPs improved pulmonary inflammation and hyperreactivity in a mouse model of asthma (Hussain et al., 2011). Treatment of the house dust mite-induced allergic airway disease model with QDs improved select cytokine production due to QDs (Scoville et al., 2019).

The effect of hyaluronic acid (HA) decorated, ferulic acid (FA)-loaded chitosan (CS) NP (FACHA) aerosolized by vibrating mesh nebulizer as a strategic combination of drug, nanocarrier, and delivery device for effectual asthma control was explored. FACHA displayed a globular structure with proper size (164.2 ± 9.7 nm) and mass median aerodynamic diameter (MMAD) of 1.81 ± 0.15 μm, indicating effective drug deposition. *In vivo* inhalation and toxicology evaluation verified safety, and FACHA prophylactically ameliorated inflammation, airway hypersensitivity, and remodeling in ovalbumin (OVA)-induced mice model of asthma (Dhayanandamoorthy et al., 2020).

The liposomal budesonide treatment in asthmatic mouse models increased efficacy in the reduction of inflammation and showed fewer side effects compared to steroids (Konduri et al., 2005). *In vivo* studies of aerosolized liposomal formulation of SBS also revealed greater drug retention time and anti-inflammatory and anti*-*asthmatic effects compared with the free drug (Chen et al., 2012).

The administration of liposomes (composition: dioleoylphosphatidylcholine and cholesterol) also reduced IL-6 and stimulated tumor necrosis factor-α (TNF-α) in primary human nasal epithelial cells. *In vivo* studies on allergic airway inflammation induced by OVA have also shown a decline in immune responses and airway responsiveness (AHR) (Komalla et al., 2020).

The effect of bilirubin-based NPs (BRNPs) as nanomedicine for the treatment of allergic lung inflammatory disease was reported. The properties of BRNPs on Th2 (allergen-specific type 2 T-helper) immune responses were studied *in vivo* and *in vitro*. After intravenous injection, BRNPs displayed more serum concentration and a longer circulation time of BR than the intraperitoneal injection of BRNPs or unconjugated bilirubin (UCB). The anti-asthmatic properties of BRNPs were evaluated in a mice model of allergen-induced asthma. Treatment with BRNPs, in comparison to UCB, inhibited the indices of experimental allergic asthma and intensely improved Th2-related allergic lung inflammation. In agreement with these results, BRNPs also decreased Th2 cell populations and the expression of associated cytokines by antibody-stimulated CD4‏^+^ T Cells *in vitro* (Kim et al., 2017).

Encapsulated and andrographolide (AG) in NP (AGNP) were assessed for anti-asthmatic efficiency by oral/pulmonary delivery. AGNP showed 5.47% drug loading with a sustained drug release *in vitro*. Pharmacokinetic results indicate improved AG bioavailability upon AGNP administration by pulmonary route. Cell numbers; IL-4, IL-5, and IL-13 levels in BALF; and serum IgE content were significantly decreased after administration of AGNP. AGNP-mediated inhibition of NF-κβ was also meaningfully higher compared to free-AG. Furthermore, results displayed improved remedial efficiency, and AGNP effectively controlled mild and intense asthma, especially when administered by inhalation. Cell numbers and IL-4, IL-5, and IL-13 levels in the BALF were also significantly reduced by AGNP (Chakraborty et al., 2019).

The capability of an intranasal treatment with NPs targeting IL4Rα to regulate lung inflammation in OVA-sensitized mice was tested. The properties of NPs treatment on the activation of lung inflammatory cells and their capability to proliferate and generate cytokines were identified by fluorescence-activated cell sorting (FACS) examination. Treatment with the anti-IL4Rα NPs meaningfully reduced proinflammatory cytokine expression and release in the BALF and lung tissue. In addition, the numbers of lung tissue lymphocytes, neutrophils, and eosinophils were reduced. Anti-IL4Rα NPs deactivated CD4 and CD8 T cells in lung tissue and inhibited their capacity to generate proinflammatory cytokines meaningfully higher than treatment with free anti-IL4Rα. Moreover, it induced a sustained low level of lung inflammation for 1 week following the last instillation compared to the treatment with free anti-IL4Rα antibodies (Halwani et al., 2016).

The remedial properties of vasoactive intestinal peptide (VIP) when conjugated to *a*-alumina NP (α-AN) to inhibit the enzymatic degradation of VIP in the respiratory system in mice were investigated. The number of eosinophils, serum IgE level, Th2 cytokines, and AHR were reduced by *a*-AN-VIP, which was more noticeable than VIP alone. These findings show that *a*-AN-VIP could be regarded as an actual nanomedicine for the treatment of asthma (Athari et al., 2016).

The potential of nasal-instilled AuNPs to inhibit allergen-stimulated asthma in a murine model showed that in both mice strains (Swiss-Webster, outbred) and A/J (inbred), AuNPs prevented the allergen-induced gathering of inflammatory cells along with the fabrication of both proinflammatory cytokines and reactive oxygen spices (ROS). In A/J mice, known as genetically asthma-prone animals, instilled AuNPs inhibited mucus generation, peribronchiolar fibrosis, and airway hyperreactivity triggered by allergen provocation (Barreto et al., 2015). Treatment with CuONPs in the respiratory structure of BALB/c mice displayed toxic effects, which were related to MAPK phosphorylation. Moreover, CuONP exposure intensified the progress of asthma (Park et al., 2016).

The immune-remedial properties of recombinant *Caryota mitis* profilin (rCmP)-loaded PLGA NPs and the principal mechanisms involved were investigated. The rCmP-loaded PLGA NPs prevented the production of specific IgE and secretion of the Th2 cytokine interleukin-4; enabled the production of particular IgG2a, the secretion of the Th1 cytokine interferon-gamma (INF-γ), the ratio of Th1/Th2; and improved allergic signs (Xiao et al., 2013). The effect of encapsulation of LPS into PLGA NPs administered by sublingual (SLIT) in asthmatic BALB/c mice was studied. Treatment with LPS-PLGA (200 nm diameter) meaningfully enhanced the cytokine levels. Histological and BALF analysis showed a reduction of eosinophilic and other inflammatory cell infiltration. Therefore, LPS-PLGA NPs can efficiently shift Th1 to Th2/Treg (Khakzad et al., 2019). In another study, the mice were treated with interferon-γ (IFN-γ) pDNA-loaded chitosan nanoparticles intra-nasally. The internalization was increased significantly by macrophages and bronchial epithelial cells. It appears that the AHR was reduced, and the lung morphology was normalized, perhaps via signal transducer and activator of transcription 4 (Kumar et al., 2003).

Grape seed-solid lipid nanoparticle (SLN) administration (30 µg/25 g/wt. 1, 2, 3, and 6 days) in C57BL/6 male mice lungs was more effective than grape seed alone in attenuating asthma process. Moreover, SLNs showed a better role in oxidative stress, suppressing airway epithelial cells, indicating the potential treatment of SLNs, drug efficacy, and drug delivery to ameliorate asthma response (Castellani et al., 2018).

**FIGURE 5:**
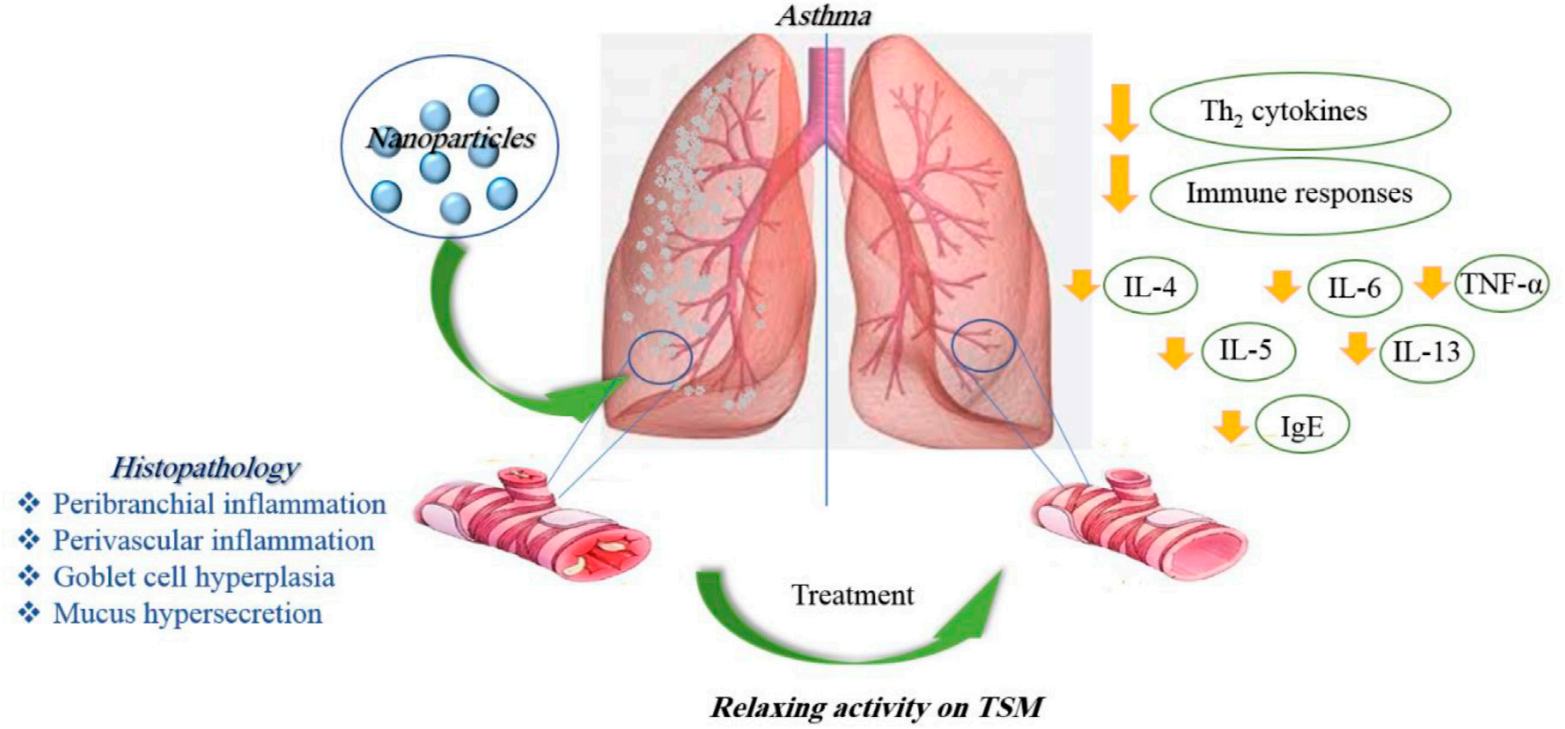
Molecular mechanisms involved in the effects of nanoparticles on asthma.

In general, *in vitro* studies have shown a reduction of inflammation in lung tissues in asthma and elucidated some molecular mechanisms due to the application of nanoparticles. *In vivo* studies, while confirming the results obtained from the *in vitro* studies, showed higher stability and treatment duration of nano-drugs than ordinary drugs. Table 1 illustrates the effects of various nanoparticles on asthma. Figure 5 demonstrates the molecular mechanisms involved in the effects of nanoparticles on asthma.

**TABLE 1:** Effects of various nanoparticles on asthma and COPD.

Study type	Study design	Dose and duration	Effect	Ref.
***In vitro***	Aerosolized *R*-TBH liposome formulation	1 mg/ml, 2 min	Prolonged anti-asthma effect	Li et al. (2018b)
	SBS dry powder inhalation	200 μg aerosolized, 14 h	Prolonged *in vitro* drug release	Honmane et al. (2019)
			↑ Bioavailability of SBS (↑hepatic metabolism and ↓ half-life)	
	TiO_2_ or AuNPs in a murine model of asthma	0.4 mg/mL or 0.8 mg/kg, 16 days	↓ AHR, BALF cell and cytokine, lung histology, serum IgE	Hussain et al. (2011)
	Solid lipids for delivering oligomeric *proanthocyanidins* from grape seed extract	2.5, 5, and 10 μM, 24, 48 h	High cellular uptake and retention time, ↓ ROS	Castellani et al. (2018)
			↓ Inflammatory responses	
	LPN for treatment of COPD	N/P ratio (N/p ¼ 1, 2, 4, 8, and 16), 1 h	↓ Steroid resistance in COPD models	Chikuma et al. (2020)
			↓ IL-8	
	Cadmium oxide nanoparticle effects on COPD	1 μg/ml, 24 h	Facilitated post-translational citrullination of proteins in COPD	Hutchinson et al. (2018)
	ZnO-NP toxicity in COPD patient samples	10, 20, and 40 μg/ml, 6 h	↑ Expression of tumor suppressor protein p53, Ras p21	Kumar et al. (2015)
	CaP/PLGA NP-mediated siRNA delivery in	50 μL, containing 5 μg siRNA	↓ Inflammatory effects	Frede et al. (2017)
			↓ CCL-2, IP-10, and IFN-γ	
	DNA damage protection by nanoforms of quercetin	25 μM, 2 h	↓ Oxidative stress	Habas et al. (2018)
			↑ CAT mRNA expression	
***In vivo***	Al_2_O_3_ NP treatments for COPD	0, 0.4, 2 mg/ml, 24 h	↓ PTPN6	Li et al. (2017)
			Phosphorylation of STAT3 lung inflammation	
	ZnO-NP treatments in COPD samples	10, 20, and 40 μg/ml, 6 h	↑ Expression of tumor	Kumar et al. (2015)
			↓ Protein p53, Ras p21, JNKs	
	DIN for the delivery of siRNA to lung vasculature	2.5 mg/kg Tie2 siRNA after 3 days	↓ Proinflammatory cytokines	Khan et al. (2015)
	LLC for therapeutic intervention in asthma	1 and 5 μg/ml, 24 h	↑ Patient compliance	Ng et al. (2018)
			↓ Proinflammatory markers	
	QDs in HDM-induced allergic airway disease in mice	100 μg, 140 ng, 10 days	↑ Select cytokine production	Scoville et al. (2019)
	CuONPs-exposed BALB/c mice	25, 50, and 100 lg/kg	↓ AHR, inflammatory cell, cytokines, ROS, MAPK	Park et al. (2016)
			↑ MAPKs phosphorylation	
	Lungs targeted using IV. Inj of NPs	10 mg/kg, 4 weeks	Significant inhibition of MMPs in the lungs	Parasaram et al. (2016)

*R*-TBH, R-terbutaline hydrochloride; SBS, nanoliposomal salbutamol sulfate; TiO_2_, titanium dioxide; AHR, airway hyperreactivity; LPN, lipid–polymer nanoparticle; BAL, bronchoalveolar lavage; GNPs, gold nanoparticles; DIN, dendrimer-inspired nanomaterials; LLC, liposomes loaded with curcumin; QDs, quantum dots; HDM, house dust mite; CuONPs, copper oxide nanoparticles; ROS, reactive oxygen species; Dur, duration; MMPs, matrix metalloproteinases; CaP, calcium phosphate.

### 4.2 Chronic obstructive pulmonary diseases and chronic bronchitis

COPD is a chronic inflammatory lung disease that causes airway obstruction. Its symptoms include difficulty breathing, coughing, and increased mucus secretion. This disease is caused by long-term exposure to irritating or airborne particles, which are often caused by cigarette smoke (Alqahtani, 2022). Airways were narrowed, and their flexibility was reduced in COPD, resulting in air trapping in the lungs (Alqahtani, 2022). Nanomaterial drugs showed therapeutic potential against various respiratory disorders as they are required in small quantities and can be effectively targeted to diseased tissue microenvironment, hence having minimal side effects (Saxena et al., 2022). The effects of nanoparticles on COPD were shown in different *in vitro* and *in vivo* studies.

#### 4.2.1 *In vitro* studies

In an earlier study, COPD cells (A549) were treated with a microRNAs (miRNAs)-loaded system using a polyester-based drug delivery platform, poly(glycerol adipate-co-ω-pentadecalactone), where it was cationic and made better surface interaction with the negatively charged cell membrane. The process of preparing nanoparticles was a single emulsification-solvent evaporation technique using cationic precursors. The *in vitro* drug delivery method was used to investigate the release profile of miR-146a. The results indicated successful miR-146a delivery to lung cells. It appears that the RNA efficacy remained even after the degradation of nanoparticles (Mohamed et al., 2019).

The potential effect of lipid–polymer nanoparticles (LPNs) for the treatment of COPD was studied. LPNs (N/p = 1, 2, 4, 8, and 16, 72 h) composed of a poly(lactic acid)/Mn-porphyrin dimer/and a cationic lipid (DOTAP) showed an actual tool against COPD. LPNs ameliorated the steroid resistance in COPD models *in vitro*, as proven by the decreased expression levels of IL-8 (Chikuma et al., 2020). Cadmium is a heavy metal that does not have a known physiological function and is often considered a toxicant (Taghavizadeh Yazdi et al., 2021). Cadmium nanoparticles as a potential cause of citrullination in COPD were shown by the effect of cadmium oxide nanoparticle (CdO NPs, 1 μg/ml, for 24 h) exposure, which facilitated post-translational citrullination of COPD proteins (Hutchinson et al., 2018). To explore differences in the recovery of exhaled NPs in subjects with COPD and never-smoking controls, the specific and local modulation of the inflammatory response by calcium phosphate (CaP)/PLGA nanoparticle-mediated siRNA (50 μL, containing 5 μg siRNA) delivery showed a promising approach for the treatment of inflammatory disorders of the lung. The NPs protect the siRNA from the environment and can be used to decrease gene expression of CCL-2, IP-10, and IFN-γ and, thereby, abrogate inflammatory responses in the lung (Frede et al., 2017). DNA damage protection by nanoforms of quercetin (25 μM, 2 h) in lymphocytes of patients with COPD exposed to the food mutagen 2-amino-3-methylimidazo (Yang et al., 2020) quinolone (IQ) was demonstrated. The ability of flavonoids to reduce oxidative stress and induce human protective enzyme systems, especially an increase in catalase mRNA expression, causes this protection (Habas et al., 2018).

#### 4.2.2 *In vivo* studies

Several drug formulations containing organic polymer-based NPs show more effective results than metallic NPs against COPD *in vivo* (Saxena et al., 2022). Al_2_O_3_ NPs (0, 0.4, 2 mg/ml, 24 h) exposure suppressed PTPN6 and phosphorylation of STAT3. Rescue of PTPN6 expression or application of a STAT3 inhibitor efficiently protected murine lungs from inflammation, as well as, in part, from the induction of COPD-like effects (Li et al., 2017). The majority of the studies concerning ZnONPs toxicity have been performed. The results showed a significant and dose-dependent increase in the expression of tumor suppressor proteins p53, Ras p21, and JNKs (Jun-N-terminal kinase) in blood samples of 31–85-year-old COPD patient samples after 6 h of ZnO treatment at the 10, 20, and 40 μg/ml concentrations (Kumar et al., 2015). Targeting of nanoparticles to damaged elastin in the lungs and a controlled release of doxycycline played an important role in reducing the MMP activity levels in rats that received single bovine serum albumin (DOX-BSA, 10 mg/kg, for 4 weeks) NP injection. An obvious advantage of such treatment is the requirement for lower dosages. Targeted single-dose delivery of doxycycline NPs significantly inhibited matrix metalloproteinases (MMPs) in the lungs for up to 4 weeks *in vivo* (Parasaram et al., 2016).

It was indicated that the cationic polymeric nanoparticles could be used successfully for drug delivery of miRNAs to the negatively charged cell membranes in COPD and lung diseases. Solid lipids prepared to deliver aerosolized oligomeric proanthocyanidins from grape seed extract (15 μl, 2.5, 5, and 10 µM) were used to treat COPD in mice. The results exhibited high cellular uptake and retention time, leading to the reduction of ROS and stability at 48 and 72 h compared with the free grape seed extract in both *in vitro* and *in vivo* settings. It also reduced the inflammatory responses in airway epithelial cells (Castellani et al., 2018).

**FIGURE 6:**
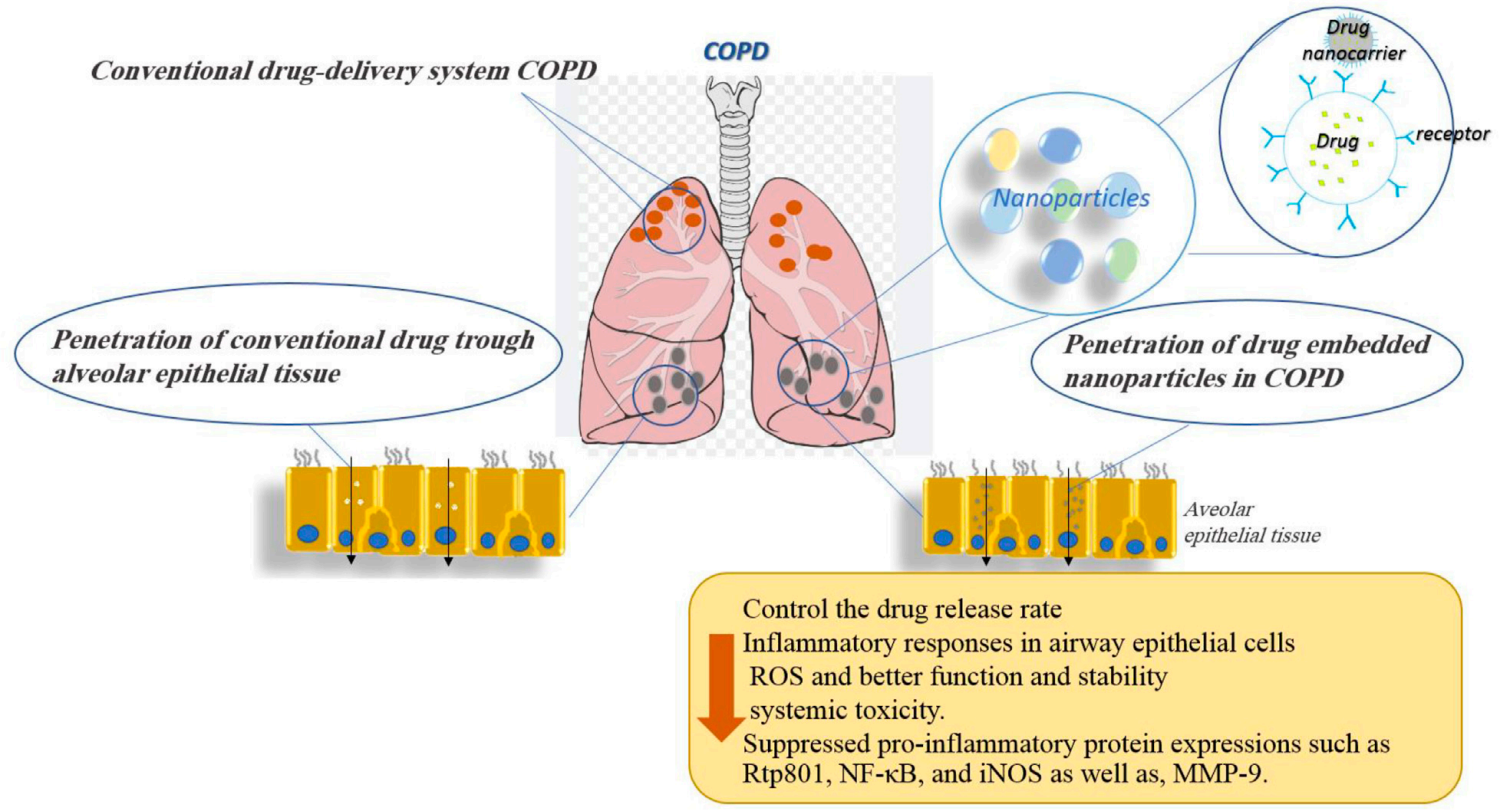
Molecular mechanisms involved in the effects of nanoparticles on COPD and pulmonary fibrosis.

In summary, the use of nanoparticles as drug delivery vehicles in COPD may prove beneficial for NPs as they could provide sustained drug release, overcome airway defenses, and target diseased cells or tissues. Indeed, the nanoparticle-based drug was shown to control the drug release rate and allowed therapeutic doses to be maintained in the lungs for longer periods while decreasing systemic toxicity. The effects of various nanoparticles used for the treatment of COPD disorders are shown in Table 1. Figure 6 demonstrates the molecular mechanisms involved in the effects of nanoparticles on COPD and pulmonary fibrosis (PF).

### 4.3 Pulmonary fibrosis and noxious agent-induced lung disorders

Idiopathic pulmonary fibrosis (IPF) is a chronic interstitial lung disease characterized by fibrosis, inflammation, and destruction of lung architecture. Damage to the alveolar epithelium and abnormal wound repair are theorized to be key factors in the development of this disease (Mei et al., 2022). Most often, the cause of pulmonary fibrosis is unknown, which is called idiopathic pulmonary fibrosis (Glass et al., 2022). Various nanoparticles showed therapeutic effects on PF in different *in vitro* and *in vivo* studies.

#### 4.3.1 *In vitro* studies

Nanostructured lipid carriers exhibiting high encapsulation efficiency (over 90%) and exceptional release profiles have the potential to treat cystic fibrosis (Moreno-Sastre et al., 2016). The administration of liposomal amikacin via inhalation for the treatment of cystic fibrosis decreased the bacterial density (Garbuzenko et al., 2014). Polyethylene glycol density and doxorubicin (DOX) were payloads on the interaction of PAMAM dendrimer with an *in vitro* pulmonary epithelium model (Calu-3). The results showed enhanced DOX transport across the pulmonary epithelium and indicated high final particle fractions (>82%) of aerosol formulations (Zhong et al., 2017).

The effect of QD containing a cadmium-selenide (CdSe) core with a zinc sulfide (ZnS) shell functionalized with carboxyl or amine terminal groups in the lung of rats was studied. Lung injuries and inflammation, such as reduced LDH, albumin, and monocyte chemotactic protein-1 (MCP-1) in the BALF, showed dependently improved concentration, which on the seventh day and for 14 hours post-exposure for both forms of QD. Both QDs missed their fluorescent assets and destabilized after 1 week in the lung. No Cd was identified in the liver, spleen, heart, brain, or blood at any time point. Cd appeared in the lung-associated lymph nodes (LALN) and kidneys beginning at 1–2 weeks after exposure (Roberts et al., 2013).

Administration of Si/SiO_2_ QDs on cell redox homeostasis in MRC-5 cells showed a time-dependent reduction in cell viability and biomass due to exposure to QDs. Si/SiO_2_ QDs showed a noteworthy influence on the intracellular distribution of glutathione. Si/SiO_2_-QDs also stimulated protein oxidation and actin S-glutathionylation (Stan et al., 2014). In the other research, male Sprague–Dawley rats were exposed to CeO_2_ or CeO_2_ coated with a nanolayer of amorphous SiO_2_ (aSiO_2_/CeO_2_) by a single intratracheal instillation. The results show that the CeO-NPs induce PF, matrix metalloproteinase-9 (MMP-9), and tissue inhibitor of MMP, but SiO_2_ coating reduced CeO_2_-induced inflammation, phospholipidosis, and fibrosis. Therefore, SiO_2_ coating showed a protective effect on CeO_2_-induced PF (Ma et al., 2015).

The neutrophils in a rat blood sample were stimulated with different concentration gradients of ZnO-NPs (0, 5, 10, 15, and 20 mg/L). The expression levels of the relevant inflammatory factor, such as IL-8, were examined. The results indicated that ZnO-NPs are capable of inducing neutrophil autophagy and regulating ZnO-NPs-induced neutrophil inflammatory response (Zhang et al., 2023).

#### 4.3.2 *In vivo* studies

Treatment of acute lung injury induced by LPS in mice with nanoemulsions containing pequi oil-oleic acid (20 mg/kg), reduced myeloperoxidase (MPO), TNF-α, IL-1β, IL-6, MCP-1, and KC levels and pulmonary leukocyte accumulation if administered orally, which can be a substitute strategy to treat respiratory inflammatory disorders (De Sá Coutinho et al., 2020s).

Complexation of small interfering RNA (siRNA) resulted in greater cellular uptake and enhanced *in vitro* silencing efficiency of TNF-α in LPS-activated mouse macrophage cell line RAW264.7, compared to morpholinium-containing dendriplexes due to higher pKa values leading to improved protection against enzymatic degradation and a higher cellular uptake. In an *in vivo* LPS-induced murine model of acute lung injury, nasal administration of siRNA (2.0 mg/kg in 30 μl, 24 h prior to administration of LPS) also showed a strong potential of phosphorus dendriplexes in lung delivery of siRNA for treating inflammatory lung diseases (Bohr et al., 2017). Administration of QD (6 μg/kg by oropharyngeal aspiration) induced acute lung inflammation, but lung mechanics were improved by QD exposure in A/J mice (Scoville et al., 2018). The inhaled tacrolimus (Tac, 60 μg/mouse) albumin NPs revealed significant anti-fibrotic efficiency in mice with bleomycin-induced PF, which was much better than the effectiveness resulting from intraperitoneal administration of Tac based on histopathological results (Seo et al., 2016). Thymoquinone (TQ)-PLGA-PVA-NPs could attenuate bleomycin-induced PF by inhibiting lung inflammation and suppressing bleomycin-induced oxidative stress. The IL-10 level was significantly reduced in rats treated with bleomycin + TQ-PLGA-PVA-NPs. In addition, the TGF-B1 level was significantly increased in bleomycin-treated rats compared with their levels in TQ-PLGA-PVA-NP-treated rats (Saghir et al., 2019).

Exposure of mice to nano-Co (50 μg) induced acute lung inflammation and lung injury indicated by an increased number of neutrophils, CXCL1/KC level, LDH activity and total protein level in the BALF, and neutrophils. Macrophage infiltration in the alveolar space and interstitial tissues, as well as immunostaining of cell proliferation markers, Ki-67 and PCNA, and the DNA damage marker, γ-H2AX, in bronchiolar epithelial cells and hyperplastic type II pneumocytes in the lungs, 7 after exposure was observed. In addition, interstitial fibrosis and inflammatory cell infiltration in the alveolar septa were observed 4 months after exposure. Moreover, nano-Co caused an increased level of 8-OHdG in the genomic DNA of mouse lung tissues. The results indicated the induction of oxidative stress, lung inflammation and injury, and cell proliferation due to nano-Co and showed the potential health effects of nanoparticle exposure (Wan et al., 2017).

Intratracheal instillation of NiO NPs (50, 100, and 200 cm^2^/rat) was compared to NiCl_2_ (171.1 μg) in an ovalbumin-induced allergic airway inflammation model. The results showed that acute neutrophilic inflammation and eosinophils were recruited 3 and 4 days after NiO NP instillation. However, the induction of inflammation was similar to that of NiCl2 24 h after instillation (Lee et al., 2015). It was reported that silica nanoparticles (SiNPs) induce PF by enhancing autophagosome accumulation and apoptosis *in vivo* and *in vitro* (Zhao et al., 2019). In addition, CuONPs aggravated pulmonary inflammation in a dose-dependent way. CuONPs induced apoptosis of epithelial cells, which was partially caused by increased ROS. In addition, CuONP exposure promoted collagen accumulation and expression of the progressive fibrosis marker *a*-SMA in the lung tissues, indicating that CuONP inhalation could induce PF in C57BL/6 mice (Lai et al., 2018). Progressive massive pulmonary fibrosis, diffuse interstitial fibrosis, and collagen accumulation involved in the development of pulmonary alveolar proteinosis following nano-indium-tin oxide nanoparticles (ITO) exposure in rats were shown (Liu et al., 2022). Chang et al. (2017) reported extensive lung fibrotic injury in histological investigation and enhanced content of hydroxyproline, collagen types I and III in rat lung tissue exposed to nano-nickel oxide (NiO, 0.015, 0.06, and 0.24 mg/kg; 6 weeks). This study suggested that PF induction by NiO-NPs may be related to TGF-β1 activation. Carbon black nanoparticles (5 mg/m^3^ and 30 mg/m^3^; 14, 28, and 90 days) also induced PF in rats, which might be linked with persistent lung inflammation through NLRP3 inflammasome activation (Zhou et al., 2020).

The reviewed articles published on the effects of nanoparticles on PF indicated improvement effects of some nanoparticles on PF. However, other studies showed the induction and aggravation of PF by other nanoparticles. Table 2 shows the effects of various nanoparticles on lung fibrosis. Figure 6 indicates the molecular mechanisms involved in the effects of nanoparticles on COPD and pulmonary fibrosis.

**TABLE 2:** Effects of various nanoparticles on lung fibrosis, lung infection, and noxious agent-induced lung disorders.

Study type	Study design	Dose and dur.	Finding	Ref.
***In vitro***	PEG and DOX payload interaction with PAMAM	30 µl DOX or 50 nM 5.5 h	↑ DOX transport	Zhong et al. (2017)
			High aerosol particle fractions (>82%)	
	OSI-420-QDs in NSCLC cell lines	400 μL QDs (1 mg/ml) for 72 h	Significantly better efficacy than pure drugs in all tested cell lines	Kulkarni et al. (2019)
	QD-CdSe-ZnS	12.5, 5.0, or 1.25 μg/rat on days 0, 1, 3, 5, 7, 14, and 28	Dose-dependent lung injury and inflammation	Roberts et al. (2013)
			Lose lung fluorescent and destabilize after 1 week	
	Si/SiO_2_ QDs on cell redox homeostasis MRC-5 cells	25 and 200 lg/ml for up to 72 h	↓ Cell viability and biomass	Stan et al. (2014)
			Impact on the intracellular distribution	
			Protein oxidation and actin S-glutathionylation	
	nAg and nZnO + CA, TA, and FA in BALF and Gamble solution	0, 3, 10, 30, 100, and 300 mg after 24 h	↑ Aggregation of nAg and nZnO	Zhong et al. (2019)
			Release Zn^2+^ promoted by CA and Ag^+^	
***In vivo***	QD-stimulated in C57BL/6J and A/J mice	6 μg/kg Cd =	QD-induced acute lung inflammation	Scoville et al. (2018)
			Impacted lung mechanics in A/J mice only	
	Cobalt nanoparticles exposed mice	50 μg per at day 1, 3, 7 and 28	↑ BALF neutrophils, CXCL1/KC, LDH and total protein, alveolar space and interstitial tissues neutrophils and macrophages infiltration	Wan et al. (2017)
			↑ Ki-67, PCNA, and γ-H2AX	
	NiO-NPs- exposed Wistar rats	50, 100, and 200 cm^2^/rat for 1, 2, 3, and 4 days	Induced neutrophilic inflammation	Lee et al. (2015)
			↑ Intracellular eotaxin and LDH in alveolar macrophages and normal lung tissue lysis	

PEG, poly(ethylene glycol); DOX, doxorubicin, BALF; bronchoalveolar lavage fluid; PAMAM, poly(amidoamine) dendrimer; OSI-420, desmethyl erlotinib; NSCLC, non-small cell lung cancer; CdSe, cadmium-selenide; ZnS, zinc sulfide; CA, citric acid; TA, tartaric acid; FA, fulvic acid; ALF, artificial lysosomal fluid; Dur, duration.

### 4.4 Lung infections (viral, microbial, parasites, and fungi)

Serious lung infections, such as pneumonia, tuberculosis, and viral lung infections, are serious lung insults and can be life-threatening (Cai et al., 2022). Lung infections involve most of the air sacs and the airways that transmit air to the lungs to a lesser extent (Cai et al., 2022). A variety of treatments or diagnostics have been utilized to manage lung infections, but the emergence of drug-resistant bacteria and the adverse effects experienced once they reach the lung environment have made treatment more difficult (Muhammad et al., 2022). The need for new strategies is felt more than before to control the activities of bacteria. In this field, a promising approach can be the use of nanomaterial science, and the knowledge that is expanding in the field of nanomaterials synthesis should be considered as a global strategy (Muhammad et al., 2022).

#### 4.4.1 *In vitro* studies

Various studies have indicated the effects of NPs on lung infections. To study the anti-tubercular drug, bedaquiline (BDQ) of chitosan nanoparticles (NPs) with an optimized batch showed a particle size of 109.7 ± 9.3 nm and a zeta potential of 36 ± 2.1 mV. *In vitro* deposition studies on non-viable cascade impactors have shown similar toxicity compared to conventional drugs. The most intriguing advantages of NPs were loading capacity, surface properties, stability in enzymes, and excellent pharmacokinetics (Rawal et al., 2018).

*In vitro* microbial studies displaying the dose-dependent manner effects of the complex of phytosome loaded drug delivery of gingerol (PG) on the respiratory infective bacterium, which verified the bactericidal properties (Singh R. P. et al., 2018).

In the United States National Library of Medicine, the amikacin-loaded liposomes were used to treat lung infection to non-tuberculous mycobacteria that have increased the efficacy, tolerance, and safety and shown a promising future for treatment, especially systemic infections (clinicaltrials.gov). Liposomal amikacin was more successful than free amikacin and can be administered by inhalation under the trade name Arikace^TM^ (Meers et al., 2008). These structures seem to be simply aerosolized and have a noteworthy result on delivery in cell culture. Their capability to better intracellular delivery *in vivo* will be affected by several factors, resulting in lung surfactant on the constancy of the structures and their rate of clearance. The fate of liposomes in the lungs was investigated, and their residence time in the lungs was found to be dependent on the ventilation degree and their structure (Cryan et al., 2006).

Calcifediol (25(OH) D) in liposomes was encapsulated to enable aerosolization for the capability to inhibit pulmonary infection by *Pseudomonas aeruginosa*, which inhibits *Pseudomonas* infection in human bronchial epithelial cells (Castoldi et al., 2017).

A type of silica nanoparticles loaded with anticancer drug doxorubicin (DOX) and antimicrobial peptide HHC36 (AMP) (MSN@DOX-AMP) significantly treated bacterial infection and eliminated *in situ* lung tumors in a commensal model. Meanwhile, MSN@DOX-AMP encapsulated DOX and AMP were highly efficient via a combined strategy of physical absorption and exhibited hemocompatibility and biocompatibility (Ma et al., 2023). Strong interactions between HA@PLGA-polymyxin B (PMB) NPs with electrical, particle size, and hydrophilicity properties and mucus resulted in more drugs entering deeper into the lung compared to the free drug PMB *in vitro* (Wu et al., 2022).

#### 4.4.2 *In vivo* studies

The pharmacokinetics of the NP formulation of BDQ were compared with the conventional dry powder inhaler and oral drug solutions *in vivo*. The results did not show a better safety profile of NPs compared to conventional DPI and oral solution, but pharmacokinetic studies indicated higher concentrations of BDQ in lungs via developed formulation (Kim et al., 2019). *In vivo* work revealed that the complex of PG ] displayed a noteworthy sustained release profile and improved the oral absorption of gingerol. The hematological characteristics revealed sustained anti-bactericidal and anti-inflammatory properties in bacterial organisms, causing respiratory infections (Chanda et al., 2010). The conjugated form of azithromycin with PAMAM dendrimer with improved membrane penetration was successful against *P. aeruginosa* biofilm associated with chronic lung infection. *In vivo* results also reduced chronic lung infection and lung inflammation (Gao et al., 2020).

Mannosylated solid lipids in the treatment of tuberculosis have increased the rifampicin uptake in macrophages and drug therapeutic efficacy and lowered drug toxicity in J774 murine macrophage cell lines and rats. Similar to the information reviewed in the liposome section, surfactants, such as mannose and synthetic ones, could enhance internalization (Maretti et al., 2019). Evaluation of the D-enantiomeric dendrimers, dG3KL and dTNS18, was studied in relation to tobramycin (Tob) to treat *P. aeruginosa*. The comparable *in vitro* activity of dG3KL and dTNS18 peptide dendrimers show *in vitro* activity comparable to Tob against both *P. aeruginosa* planktonic and biofilm cells at concentrations not toxic *in vivo* (Pompilio et al., 2018).

Mice (C57BL/6J) were treated with ONP-302-NPs (1 mg) via injection (i.v.) on day 3 after infection. The results indicated that daily particle administration from 3 dpi to either 5 or 7 dpi reduced the accumulation of monocytes within the lungs and ameliorated clinical signs during infection. In addition, ONP-302 reduced the levels of MPO, albumin, IL-6, and TNF-α in the lungs of influenza-infected mice (Kelley et al., 2022). In a mouse model of acute *P. aeruginosa* pneumonia, algae–nanoparticles effectively reduced bacterial burden and substantially reduced animal mortality (Zhang F. et al., 2022)

**FIGURE 7:**
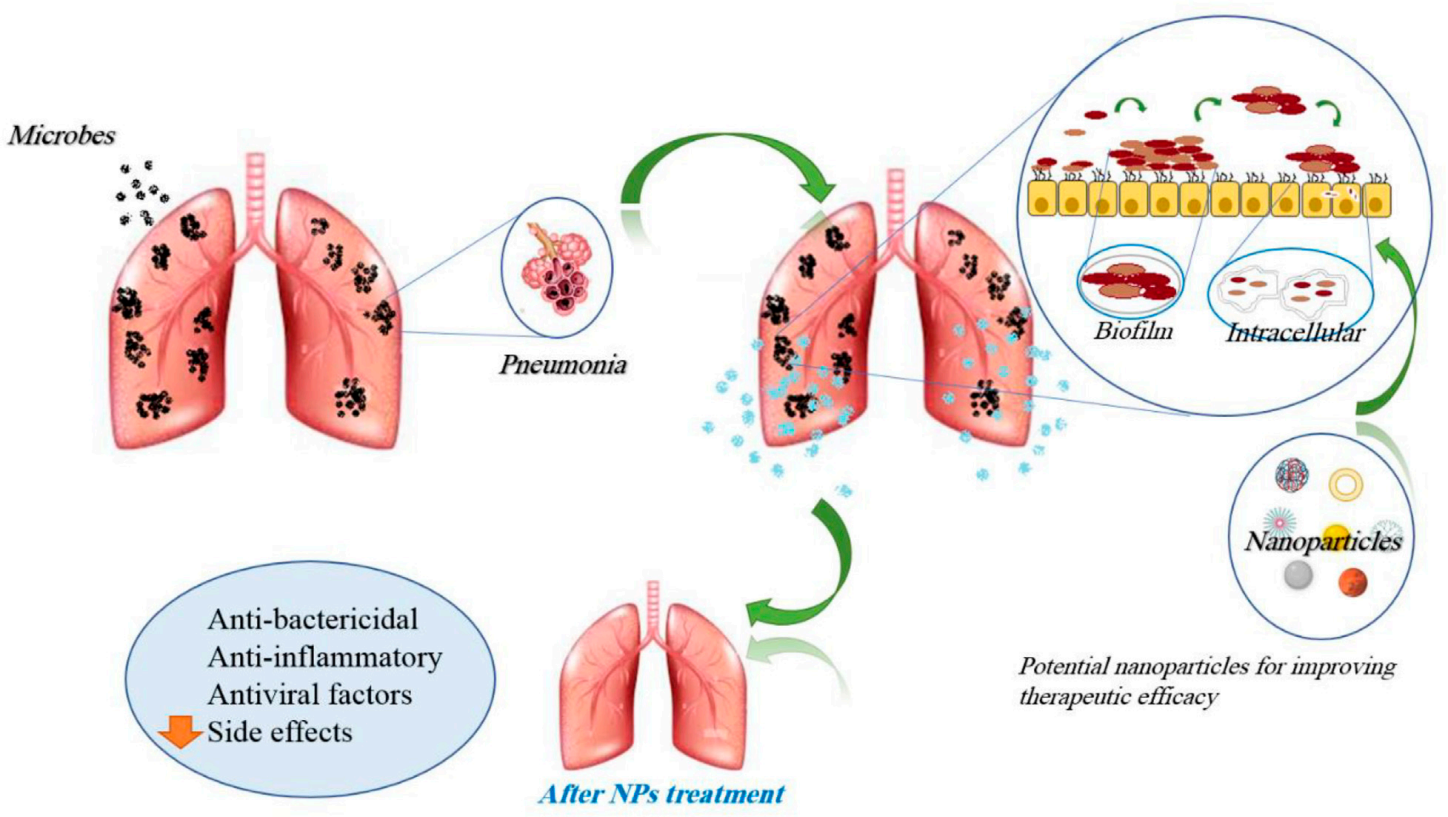
Molecular mechanisms involved in the effects of nanoparticles on lung infections.

Taken together, the administration of nanoparticle-based medicine is a promising tool for the treatment of lung infections through overcoming low local antibiotics. Additionally, the increased antibacterial activity of nanoparticle-based medicine against bacteria promises decreased doses, thus reducing their side effects. The effects of nanoparticles in lung infections are mentioned in Table 2. Figure 7 demonstrates the molecular mechanisms involved in the effects of nanoparticles on lung infections.

### 4.5 Lung cancers

The most common type of cancer in the world is lung cancer, and nanomaterials play an important role in cancer therapy (Sanaei et al., 2022). Lung cancer, characterized by the uncontrolled growth of cells in lung tissues, remains a substantial and disturbing origin of universal disease and death. Nano-therapy and regenerative medicine have continued to develop for lung biology and lung disorders (Sanaei et al., 2022). Several studies have shown the effects of NPs on lung cancer.

#### 4.5.1 *In vitro* studies

Some studies have shown the effects of NP drugs on increasing delivery to lung cancer cells and diagnosis of lung cancer. Alginate or chitosan-coated single-walled carbon nanotubes (0, 4, 8, 12, 16, and 20 μg/ml for 24 h) were used to deliver curcumin to the A549 lung cancer cells. The loading efficiency and the sustained drug release are two main advantages of the mentioned nanoplatforms.

Studies have shown the promising potential of carbon-based nanoparticles, including fullerene, carbon nanotubes, and graphene-based materials, for drug delivery purposes due to high drug loading and outstanding drug release profiles (Li et al., 2014).

Poly(3-hydroxybutyrate-co-3-hydroxyvalerate acid) (PHBV), a biodegradable and biocompatible bacteria-produced biopolymer, was applied to prepare nanoparticles containing sunitinib, a common drug used to treat lung cancer with low selectivity. This NP is a selective epithelium transport and drug internalization, with low side effects due to controlling the release profile. The MTT assay (0.5, 2, 4, 8 μg/ml NPs after 48 h) has shown comparable cytotoxic effects to sunitinib, which demonstrated the promising future of this drug delivery system (Otroj et al., 2020).

Surface-modified ZnS QDs (0, 1, 10, 50, 100 μg/l for 48 h) with adipic dihydrazide-heparin were used to deliver paclitaxel to the A549 cancer cells. *In vivo* and *in vitro* studies have shown a more controllable drug release profile triggered by acidic conditions, which could enhance drug efficiency and cytotoxicity in cancer treatment (Xie et al., 2020).

In another study, dihydropyrimidinones were formed by conjugation with carbon QDs with various ratios (25, 50, 100, 200, 300, 400, and 500 μM, 24 h). The lower toxicity against healthy cells, fluorescence trackability by bioimaging, and improved tumor-growth inhibition were obvious from the results. QDs could give the nanocomposites multi-functionalities of drug monitoring, trackability, delivery, and medical imaging altogether to the nanocarrier (S et al., 2021). However, sometimes the cytotoxicity of QDs in some cases is an issue that needs special attention (Zhang et al., 2020c). In general, the tuneability, modifiability, stability, and biocompatibility of carbon QDs and inexpensiveness are the main advantages of carbon QDs (Guo et al., 2018). The application of QDs to medical imaging can also facilitate decisions on diagnosing and staging of cancer and consequently choosing the right medication (Jin et al., 2011). Using non-small cell lung cancer biomarkers conjugated with QDs, one could also detect lung cancer metastasis in blood or lymph nodes (Wang et al., 2012).

Magnetic nanoparticles and superparamagnetic iron oxide nanoparticles (SPIONs) are the most usually employed metal oxides in therapy, diagnosis, and separation (Cedrowska et al., 2020). It has been shown that these properties may result from the increased antioxidant effects of these particles. The centerpiece for mag-force cancer therapy is also iron oxide nanoparticles (Gao et al., 2017). The small size of the particle is critical for therapy. Hence, its high density has therapeutic effects when it is injected directly into tumor cells. An external field will cause absorption of the magnetic nanoparticles by cancer cells, after which further oscillation causes drug release, hyperthermia, or cell burst (Alphandéry, 2020).

In lung cancer, metastasis could be detected with sensitive SPIONs by magnetic resonance imaging with no side effects (Wan et al., 2016).

A dendrimer-based nano-drug delivery system was designed to be capable of increasing the penetration of DOX as measured in an *in vitro* 3D lung tumor model and associating those consequences and its efficiency. More specificity for DOX to tumor cells compared to fibroblasts due to conjugation, maintenance activity of the released DOX that reduces tumor similar to free DOX was observed (Almuqbil et al., 2020). The ZnO QD-based pH-responsive drug delivery pattern (0.1, 1, 5, 10, 25, and 50 μg/ml, 48 h) for intracellular controlled release of drugs was designed. Synergistic therapy was observed due to the incorporation of the antitumor results of Zn^2+^ and DOX (Cai et al., 2016).

Several other studies indicate the therapeutic effects of NPs on lung cancer. The cytotoxicity of the alginate-coated nanotube against A549 cells was also more efficient than that of the free curcumin (Singh N. et al., 2018). A similar docetaxel-loaded chitosan-coated NPs (40 μg/ml for 1, 2, and 4 h.) led to higher drug bioavailability compared to the free drug, which has demonstrated the growth inhibition of 66% against A549 tumor cells. (Li B. et al., 2018).

Chitosan-coated graphene oxide functionalized with the hyaluronic acid (5, 10, 20, 40, 80, 160, and 320 μg/ml for 24 h) as the targeting agent led to tolerable systemic side effects and higher internalization and toxicity against A549 cancer cells compared to normal cells (Liu et al., 2018). The nanocomposite of NiO/CuO nitrogen-doped graphene oxide (5, 10, 50, 100, 150, and 200 μg/ml) has also illustrated the potential to kill A549 cancer cells (Anbu et al., 2021). This study also suggests that A549 cell killing is mediated through ROS generation induced by NiO/CuO NC.

Graphene oxide dots have recently been used as a photosensitizer in photodynamic therapy for lung cancer. It appears that dots were internalized and induced ROS, leading to apoptosis and necrosis (Shih et al., 2021). Fullerenes, another allotrope, are nanocages of carbon that take different hollow shapes from spherical to cylindrical and are very effective radical scavengers. Despite these attractive properties, carbon nanomaterials are toxic and show other problems, such as agglomeration, difficult administration routes, and particle size (Nalepa et al., 2020).

PAMAM was used to transport multiple chemotherapeutics, including cisplatin and small interfering RNAs (siRNA). Folic acid was conjugated for favorable targeting purposes against H1299 lung cancer cells. The target cells expressed folate receptor alpha. The siRNA was deposited electrostatically, and cisplatin was attached via the coordination of the amine group. It appears that the drug damages the DNA and induces apoptosis of cancer cells, which increased synergistically compared to the drugs used individually, and the toxicity also decreased due to targeting using folic acid (Amreddy et al., 2018).

Antisense oligonucleotide (2′-O-methyl-RNA)-loaded chitosan nanoparticles (0.1–2.5 mg/ml for 6 h) were used to treat A549 cancer cells via telomerase inhibition, which decreased the telomerase activity significantly (50%) and was potentially promising for the treatment of lung cancer (Nafee et al., 2012).

Recently, folate-decorated Ag–In–Zn–S QDs (50 μg ml^−1^, 24 h) were applied for targeting drug delivery of doxorubicin to A549 cancer cells. The *in vitro* analyses demonstrated that the nanoconjugate was more cytotoxic and genotoxic than the free drug. It appears that the prepared conjugate successfully inhibited the migration of cancer cells (Ruzycka-Ayoush et al., 2021).

The thiolate–Zn interaction was also used to attach a histone deacetylase inhibitor, *N*-hydroxy-*p*-(4-arylbutanamido) benzamides, to the CdSe/ZnS QDs, which formed a water-miscible nanocarrier (20 nM, 48 h) that was employed to treat A549 and H1299 cancer cells. Investigation of the apoptosis cycle showed the breakdown of the cell cycle at the G2*-*to*-*M transition while increasing the acetylation of p53 and tubulin, killing cancer cells, or inhibiting tumor growth synergistically and more efficiently than the free active agent (Chen et al., 2021).

Carbon QDs conjugated with Bi_4_O_5_Br_2_ were successfully implemented for tumor photodynamic therapy against A549 cancer cells (0, 0.5, 5, 10, 25, 50, 100, and 200 μg/ml, 24 h). Compared to Bi_4_O_5_Br_2_ nanocrystals, QD nanocomposites with Bi_4_O_5_Br_2_ were water-miscible, improved the uptake of ROS generation, especially O_2_^−^ and ·OH species, and were more effective in killing lung cancer cells. Therefore, it can be used to enhance the efficiency of photocatalytic properties, biocompatibility issues, and internalization of the inorganic active materials (He et al., 2021). Carbon QDs in drug delivery systems can increase the therapeutic potency of drugs in the treatment of different cancers (Li et al., 2020).

Gold nanoparticles are possible candidates for gene therapy, drug delivery, photothermal therapy, and photodynamic therapy, especially in lung cancer and viral diseases (Mousavi-Kouhi et al., 2021b). In lung cancer, bombesin*/*GRP receptors were overexpressed; therefore, synthesizing a library of them and their conjugated forms of AuNPs would show high biocompatibility (Chanda et al., 2010). Research has shown that gold nanoparticles bind to drugs by binding to various functional groups, such as carboxyl groups. AuNPs (0.16, 0.31, 0.63, 1.25, 2.50, and 5.00 mg/ml; 24, 48, and 72 h) can enhance ROS production, sensitize mitochondrial membrane potential, and stimulate both primary and late apoptosis in lung cancer cells (Mousavi-Kouhi et al., 2021b).

The conjugated form of Toll-like receptor 4 (TLR4) with AuNPs was used as an adjunct therapy for human adenocarcinoma alveolar basal epithelial cells (A549 cells), which led to the downregulation of the expression of this receptor (Vyas and Goswami, 2019). AuNPs (20, 40, 80, and 100 μg/ml for 24 h) were found to be most effective in preventing ROS generation by TLR4 in A549 cells. RNI produced by unrestrained TLR4 signaling is recognized for the development of lung carcinogenesis, and AuNPs significantly decreased RNI production (Vyas and Goswami, 2019).

It has been reported that the cisplatin-dendrimer nanocomplex has toxic behavior against NCI-H460 lung cancer cells (Nguyen et al., 2015). Assessment of the safety profile and remedial efficiency of the anticancer factor derived from [188Re] rhenium-ligand as radioactive ligand-loaded 5th production poly-L-lysine dendrimer was performed. The results showed a safe and well-tolerated 188-Rhenium-Imdendrim brachytherapy device for lung cancers (Belhadj-Tahar et al., 2019).

Significant improvement of cellular uptake along with gene transfection efficacy of PAM-Ap/pMiR-34a NPs in cultured NSCLC cells due to aptamer conjugation was indicated (Wang H. et al., 2015). Phenethyl isothiocyanate (PEITC) and cisplatin (CDDP) co-encapsulated in a liposomal NP for the treatment of non-small cell lung cancer were previously studied. The results displayed an enhancement of NSCLC cell toxicity due to co-encapsulation of PEITC and CDDP in liposomes. Liposomal-PEITC-CDDP was more toxic against both A549 and H596 human NSCLC cell lines than against WI-38 and BEAS-2B human normal lung cell lines (Sun et al., 2019).

Gefitinib (GEB) encapsulation upon the nanoliposomes (10, 20, 30, 40, and 50 μg/ml, 24 h) for increasing the curative effect of lung cancer was designed. Improved stability, reduced particle size, and higher encapsulation efficacy in nanoliposome compound drug (GL) were observed. In addition, *in vitro*, GL had a promising drug sustained-release result. GL significantly increased the proapoptotic result on A549 cells. The ability of GET to inhibit the proliferation, migration, and invasion of tumor cells has been significantly enhanced after nanoliposome embedding modification (Hu et al., 2020).

Co-administration of curcumin and bromocriptine nano-liposomes (12–20 μM; 12, 24, and 48 h) to human lung cancer cells (QU-DB) *in vitro* showed anticancer effects by affecting apoptosis and cell viability in QU-DB cells but did not show cell toxicity properties in normal cells (HFLF-PI5) (Sheikhpour et al., 2020). Electroporation preparation of paclitaxel-in-liposome-in-bacteria (LPB) (0.62, 1.25, 2.50, and 5 μmol/L, 24 h) was performed for inhalation treatment of primary lung cancer. LPB prevented the proliferation of A549 lung cancer cells. LPE displayed the maximum anticancer effect on rat primary lung cancer with the downregulation of VEGF and HIF-1α and the improvement of cancer cell apoptosis after intratracheal administration. Bacterial formulations significantly increased the expression of immune markers (TNF-α, IL-4, and IFN-γ) and immune cells (leukocytes and neutrophils) (Zhang et al., 2020b).

The cell toxicity of InP/ZnS QDs (0.62, 1.25, 2.5, 5, 10, 20, 40, 80, and 160 μg/ml, 24, 48 h) with diverse surface clusters (NH_2_, COOH, and OH) against two lung-derived cell lines was reported. All of the InP/ZnS QDs were able to enter the cells, with greater uptake efficacy for InP/ZnS-COOH and InP/ZnS-NH_2_ at lower doses. High doses of InP/ZnS QDs initiated the reduction of cell viability, and InP/ZnS-COOH QDs and InP/ZnS-NH2 QDs seemed more toxic than InP/ZnS-OH QDs. In addition, InP/ZnS QDs developed cell apoptosis and intracellular ROS production after co-culture with cells (Chen et al., 2018).

The green fabrication of CdS QDs using tea leaf extract resulted in the inhibition of bacterial growth and displayed cell toxicity against A549 cancer cells due to CdS QDs. Production of high-contrast fluorescence images of A549 cancer cells due to CdS QDs was another result of this study. CdS QDs arrest the A549 cell growth at the S phase of the cell cycle, preventing more growth of lung cancer cells. CdS-QDs could interact with the P-part in DNA. Then, DNA-replication is inactivated, leading to the inhibition of enzyme functions, which results in loss of cell viability and undergoes cell death through apoptosis (Shivaji et al., 2018).

Black phosphorus quantum dots (BP-QDs, 0, 5, 10, and 20 μg/ml for 24 h) on lung cells (A549 and Beas-2B) induced cell toxicity and reduced oxidative stress and cell cycle arrest in two types of human lung epithelial cells. The prevention of cell uptake and restoration of the cell toxicity of BP-QDs were also shown. Oxidative stress also contributes to cell cycle arrest and cell damage caused by BP-QDs. The results showed that BP-QDs induced LDH (an indicator of cell membrane integrity) leakage from A549 and Beas-2B cells in a concentration-dependent manner (Ruan et al., 2021). Exposing A549 cells to 70 and 420 nm ZnO particles (0, 8, 10, 12, 14, 16, and 18 μg/ml, 24 h) showed dose- and time-dependent cytotoxicity due to exposure of both sizes of NPs. Neither free Zn^2+^ nor metal impurities in ZnO NP samples are the cause of cytotoxicity. The cytotoxicity of ZnO is reflected in oxidative stress, lipid peroxidation, cell membrane damage, and oxidative DNA damage (Lin et al., 2009).

Metal and metal oxide nanoparticles (ZnO, CeO, and Ag) (8–128 μg/ml; 1, 6, 24 h) in lung epithelial A549 cells caused metabolic and transcriptional responses, and the great majority of these molecular alterations were usual to both ionic and NP exposures and a characteristic of metal ion exposure. The low toxicity of CeO_2_ NPs elicited few molecular changes, displaying few signs of oxidative stress for only one of the four CeO_2_ NPs examined (Dekkers et al., 2018). Green AgNP (10–250 μg/ml; 24 h) fabrication using *Cymodocea serrulata* as a potential marine biosource displayed potential cellular toxicity in human lung cancer A549 cells (Palaniappan et al., 2015).

Biosynthesis of gold nanoparticles (AuNPs, 20–100 μg/ml, 24 h) from *Magnolia officinalis* and anticancer activity in A549 lung cancer cells efficiently induced cellular toxicity and apoptosis indicated by apoptotic gene expression. Synthesized AuNPs provoked ROS production in A549 cells, and elevated ROS directed to the fragmentation of the nucleus and depolarization of mitochondria, resulting in oxidative stress inter-ceded apoptosis. AuNPs diminished the expression of Bcl-2 and Bid and elevated the expression of Bax, Beclin-1, and caspase-3 in A549 cells (Zheng et al., 2019).

The ability of nanoconjugates of CdSe/CdS/ZnS-QDs and Dox (330 μl, 24 h) to target alveolar macrophage cells was studied. The release of Dox from the QD-Dox nanoconjugate induced apoptosis. Inflammatory injury parameters, including albumin leakage, proinflammatory cytokines, and neutrophil infiltration, were documented after the admiration of QD-Dox (Chakravarthy et al., 2011). In the presence of laser irradiation, GA-AuNPs showed considerable cytotoxicity against A549 cells. Lung tumor treatment with GA-AuNPs followed by laser exposure enhanced the apoptotic pathway. Upregulation of caspase-3, a significant reduction in the levels of the inflammatory mediator TNF-α, and angiogenesis inducer VEGF were also observed. Induction of lipid peroxidation was also reported upon treatment with GA-AuNPs (Gamal-Eldeen et al., 2017). It was indicated that QDs conjugated to desmethyl erlotinib (QD-OSI 420) showed meaningfully better efficiency than pure drugs in non–small cell lung cancer (NSCLC) cell lines, and QD-OSI 420 conjugated significantly increased cell viability compared to pure drugs with an IC_60_ of 2.5 μM in erlotinib-resistant A549 cell lines. In addition, in the 3D-SCC model of A549, QD-OSI 420 significantly reduced 3D tumor volume compared to pure drugs *in vitro*. Therefore, these results indicate that conjugating QD-OSI-420 could be considered an alternative to traditional anticancer therapy by improving intracellular drug delivery (Kulkarni et al., 2019).

#### 4.5.2 *In vivo* studies

Paclitaxel-loaded solid lipids (1.0, 10, and 100 μg/ml, 1 h) were used against metastatic mammary tumors in the lungs *in vivo* and *in vitro*. Results revealed drug accumulation in the tumor microenvironment, intracellular targeting, cellular toxicity, increased therapeutic delivery into the cytosol, and prevention of further tumor progression (Videira et al., 2012). In fact, PTX-loaded liposomes did not attain more efficiency but rather induced severe neurotoxicity, proposing the existence of a free drug. Remedial efficacy of a new cationic liposome-nano-formulated all-trans-retinoic acid (0.60 mg/kg/day for 30 days) in the mice model of lung cancer revealed that the formulation of lipo-all-trans-retinoic acid in 1, 2-dioleoyl-3-trimethylammonium-propane (DOTAP) and cholesterol in the ratio of 70:20 was the suitable carrier for ATRA in treating lung cancer (Grace and Viswanathan, 2017). As DOTAP is a cationic lipid, the interaction with a negatively charged cell surface may facilitate an effective fusion and drug delivery in the target site. The liposome-formulated (“antineoplastic” or “cytotoxic”) chemotherapy drug enhances the stability of the ATRA and delivers the drug to the target site effectively. The use of natural polysaccharides is also common to make nanoemulsions to treat different types of cancers. For example, *Brucea javanica* oil with chitosan formed a cationic nanoemulsion (505 mg kg^−1^, 28 days) that showed high colloidal stability and a higher oral bioavailability (1.6-fold more). The nanoemulsion enhanced the suppression of lung cancer compared to free oil in *in vivo* models (Liu et al., 2016). VEGF, IL-8, and Bcl-2 were downregulated, and P53 was upregulated.

#### 4.5.3 Clinical studies

The efficacy of IFN-γ nanoliposome on peripheral lymphocytes from lung cancer patients was investigated compared to healthy individuals (20 individuals in each group). The lymphocytes from lung cancer patients presented with upper DNA destruction ranked higher than those of healthy individuals. In healthy individuals, IFN-γ liposome did not stimulate any DNA destruction in the lymphocytes. It also decreased DNA destruction in the lymphocytes of lung cancer patients. IFN-γ liposome meaningfully decreased oxidative stress (Alhawmdeh et al., 2021).

In a clinical study, SLIT liposomal cisplatin was administered via inhalation for patients with lung carcinoma. The results indicated the absence of dose-limiting toxicity at the maximum delivered dose, including no hematologic, kidney, ophthalmic, or nervous toxicities (Wittgen et al., 2007).

**FIGURE 8:**
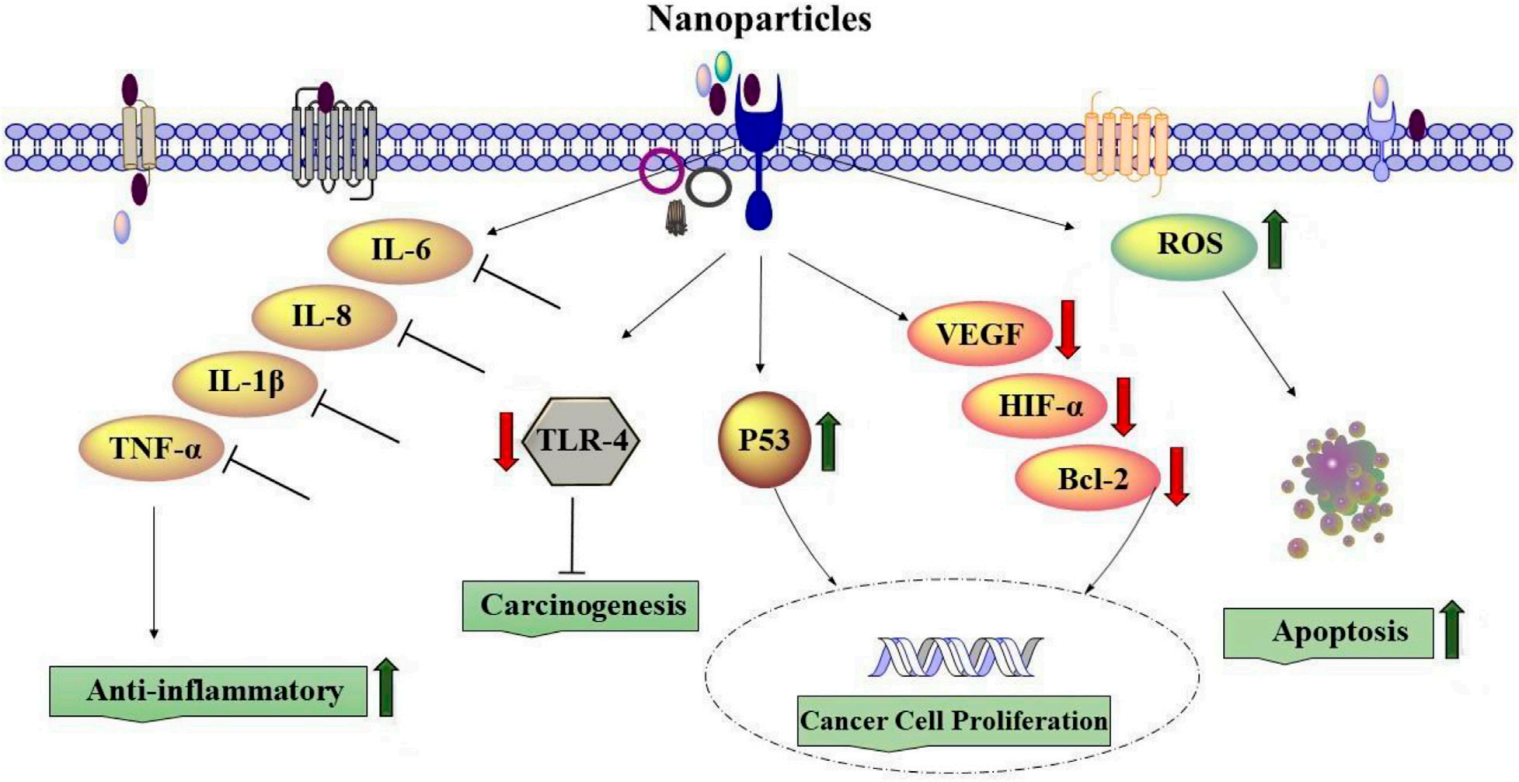
Molecular mechanisms involved in the effects of nanoparticles on lung cancer.

Overall, various NPs were developed for selective drug delivery to lung cancer and lung metastases based on understanding the biology of the tumor, the microenvironment, and the interaction between malignant cells and nanoparticles. The results showed the NP-based drug as an alternative to traditional anticancer therapy, which is more effective than ordinary drugs by improving intracellular drug delivery. The effects of nanoparticle treatment in lung cancers are documented in Table 3. Figure 8 shows the molecular mechanisms involved in the effects of nanoparticles on lung cancer.

**TABLE 3:** Effects of various nanoparticles on lung cancers.

Study	Study design	Dose and time	Finding	Ref.
***In vitro***	MDM2-exposed rats	0–100 μg/ml for 24 h	Downregulated MDM2 gene, activation of Cas-8 and PARP cleavage, ↑ cell apoptosis, inhibited tumor growth	Huang et al. (2016a)
	CS-coated CNT for delivery and Cur of A549 cells	0, 4, 8, 12, 16, 20 μg/ml for 24 h	Cytotoxicity of alginate-coated nanotube against A549 cells was also more efficient than the free curcumin	Singh et al. (2018a)
	DTX-loaded CS-coated	40 μg/ml for 1, 2, and 4 h	↑ Drug bioavailability	Li et al. (2018a)
			Inhibited A549 tumor cells	
	CS-coated GO against A549 cancer cell	5, 10, 20, 40, 80, 160, and 320 μg/ml, 24 h	↓ Systemic side effects	Liu et al. (2018)
			↑ Internalization and toxicity s	
	NiO/CuO nanocomposite nitrogen-doped GO	5, 10, 50, 100, 150, and 200 μg/ml	↑ ROS generation by NiO/CuO	Anbu et al. (2021)
			↑ Killing of A549 cancer cells	
	ASO (2′-O-methyl-RNA)-loaded CS NPs	0.1–2.5 mg/ml for 6 h	↓ Telomerase activity significantly in A549	Nafee et al. (2012)
	PHBV nanoparticles containing sunitinib	0.5, 2, 4, and 8 μg/ml NPs after 48 h	↓ Side effects	Otroj et al. (2020)
			Controlling the drug release profile	
			Comparable cytotoxic effects of sunitinib	
	Ag–In–Zn–S QDs fin A549 cancer cells	50 μg ml^−1^, 24 h	↑ Cytotoxic and genotoxic than free drug	Ruzycka-Ayoush et al. (2021)
			↓ Migration of cancer cells	
	CdSe/ZnS QD nanocarrier to treat A549 and H1299 cancer cells	20 nM, 48 h	Breakdown of the cell cycle at *G2-*to*-M* transition	Chen et al. (2021)
			↑ Acetylation of p53 and tubulin	
			Killing cancer cells or inhibiting the tumor-growth	
	ZnS QDs + ADH in A549 cancer cells	0, 1, 10, 50, and 100 μg/ml for 48 h	↑ Drug efficiency and cytotoxicity in cancer treatment	Xie et al. (2020)
	Encapsulated ZnO-QDs to poly(DL-lactide-*co*-glycolide)	7.5 μg/ml for 24 h	Selectively cytotoxic against metastatic A549 cells	Kim et al. (2020)
	DHMPs formed a conjugate with CQDs	25, 50, 100, 200, 300, 400, and 500 μM, 24 h	Inhibited tumor growth	Thangamani et al. (2021)
			Improved rug monitoring, trackability, delivery	
	Conjugated Bi_4_O_5_Br_2_ + C_2_ QDs f against A549 cells	0,0.5, 5, 10, 25, 50, 100, and 200 μg/ml, 24 h	↑ Uptake and reactive oxygen generation in cancer cells leads to killing them	He et al. (2021)
	AuNP treatment in lung cancer cells	0.16, 0.31, 0.63, 1.25, 2.50, and 5.00 mg/ml; 24, 48, 72 h	↑ ROS production	Mousavi-Kouhi et al. (2021b)
			Improved mitochondrial membrane potential	
			Apoptosis in lung cancer cells	
	Toll-like receptor 4 conjugated + AuNPs for A549 lung cancer cells	20, 40, 80, and 100 μg/ml for 24 h	Downregulation of TLR4 expression	Vyas and Goswami (2019)
			↓ RNI production by unrestrained TLR4 signaling	
	GEB encapsulation nano-liposomes for the treatment of lung cancer cells	10, 20, 30, 40, and 50 μg/ml, 24 h	Improved GL stability, encapsulation efficacy	Hu et al. (2020)
			↓ Particle size, proliferation, migration, and invasion	
			↑ Proapoptotic result on A549 cells	
	Cisplatin-stimulated NCI-H460 lung cancer cell	100 μg/ml for 48 h	Toxic effect against NCI-H460 lung cancer cell	Nguyen et al. (2015)
	Nano-DDS + ∼5.5 DOX	0.1 μM–20 μM	More specificity for DOX to tumor cells	Almuqbil et al. (2020)
			Maintenance activity of the released DOX	
	PAM-Ap/pMiR-34a NP- stimulated NSCLC	-	↑ Cellular uptake, gene transfection efficiency of NPs in cultured NSCLC cells	Wang et al. (2015a)
	LN-encapsulated PEITC and CDDP-stimulated NSCLC	-	↑ Cell toxicity to A549 and H596 human NSCLC cell lines than WI-38 and BEAS-2B HNCL	Sun et al. (2019)
	DOTAP carrier for ATRA- exposed mice	0.60 mg/kg/day for 30 days	Suitable carrier for ATRA in treating lung cancer	Grace and Viswanathan (2017)
	Cur + BR nano-liposomes	BR: 12.5–25 μM	Anticancer effects on lung cancer cells	Sheikhpour et al. (2020)
		Cur: 12–20 μM	No cytotoxicity effects in HFLF-PI5	
	Paclitaxel in the liposome model of lung cancer	24 h after 1 ml of LPB	↓ Proliferation of A549 cells, VEGF, and HIF-1α	Zhang et al. (2020b)
			↑ Effect on the rat primary lung cancer	
			↑Expression of TNF-α, IL-4, and IFN-γ	
	IFN-γ liposome peripheral lymphocyte	30 min incubation 100 U/ml	Induce DNA damage in the lymphocytes	Alhawmdeh et al. (2021)
			↓ DNA damage in lung cancer lymphocytes	
			↓ Oxidative stress caused by H_2_O_2_	
	ZnO- QD model of lung cancer	0, 0.1, 1, 5, 10, 25, 50 μg/mL, 48 h	Synergistic therapy due to the incorporation of the antitumor effect of Zn^2+^ and DOX	Cai et al. (2016)
	InP/ZnS QD-stimulated two lung-derived cell lines	0, 0.62, 1.25, 2.50, 5, 10, 20, 40, 80, and 160 μg/ml, 24 h	↑ Uptake of InP/ZnS-COOH and InP/ZnS-NH_2_	Chen et al. (2018)
			↓ Cell viability	
			↑ Toxic effect, apoptosis, intracellular ROS	
	CdS-QD-stimulated A549 cancer cells	0, 10, 20, 30, 40, 50, and 60 μg/ml, 24 h	↓ A549 cell growth at the S cell cycle phase	Shivaji et al. (2018)
			↑ Cytotoxicity on A549 cancer cells	
			↑ High-contrast fluorescence A549 cells images	
	BP-QD-stimulated A549 and Beas-2B lung cell	5∼20 μg/ml for 24 h	↓ Cytotoxicity, oxidative stress, cell cycle arrest	Ruan et al. (2021)
			↓ Cellular uptake restores the cytotoxicity of BP-QDs	
	ZnO NP-stimulated A549 lung cells	0,8, 10, 18, and 25 μg/ml in 24 h	Dose- and time-dependent cytotoxicity	Lin et al. (2009)
	ZnO, CeO, and Ag- stimulated A549 cells	8, 15, 10, and 30 μg/ml in 24 h	↑ Metabolic and transcriptional responses	Dekkers et al. (2018)
			Evidence of oxidative stress	
	Green AgNPs synthesis using *Cymodocea serrulata*	10–250 μg/ml after 24 h	Potential cytotoxicity against human lung cancer A549 cells	Palaniappan et al. (2015)
	AuNP-stimulated A549 lung cancer cells	15 and 20 μg/ml after 24 h	↑ Cytotoxicity and apoptosis by inflecting apoptotic gene expressions in A549 cells	Zheng et al. (2019)
***In vivo***	CdSe/CdS/ZnS QDs and Dox-stimulated alveolar macrophages	10 nM QD-Dox after 24 h	↑ Cell apoptosis	Chakravarthy et al. (2011)
			Improved inflammatory cytokines markers	

MDM2, mouse double minute-2; CS, chitosan; GO, graphene oxide; CNT, carbon nanotube; Cur, curcumin; DTX, docetaxel; ASO, antisense oligonucleotide; DHMPs, dihydropyrimidinones; CQDs, carbon quantum dots; ADH, adipic dihydrazide-heparin; DOTAP, 1, 2- dioleoyl-3-trimethylammonium-propane; Cas-8, caspase-8; PARP, poly(ADP-ribose) polymerase; miR-34a, microRNA-34a, into PAM-Ap/pmiR-34a NPs, S6 aptamer-conjugated dendrimer nanoparticles; PHBV, poly(3-hydroxybutyrate-co-3-hydroxyvalerate acid); AuNPs, gold nanoparticles; AgNPs, silver nanoparticles; PEITC, phenethyl isothiocyanate; CDDP, cisplatin; GEB, gefitinib; GL, nanoliposome compound drug; LN, liposomal nanoparticle; LPB, liposome-in-bacteria; Cur, curcumin; Dur, duration; BR, bromocriptine; DOXeq, DOX-equivalent; HNCL, human normal lung cell line.

## 5 Discussion

**FIGURE 9:**
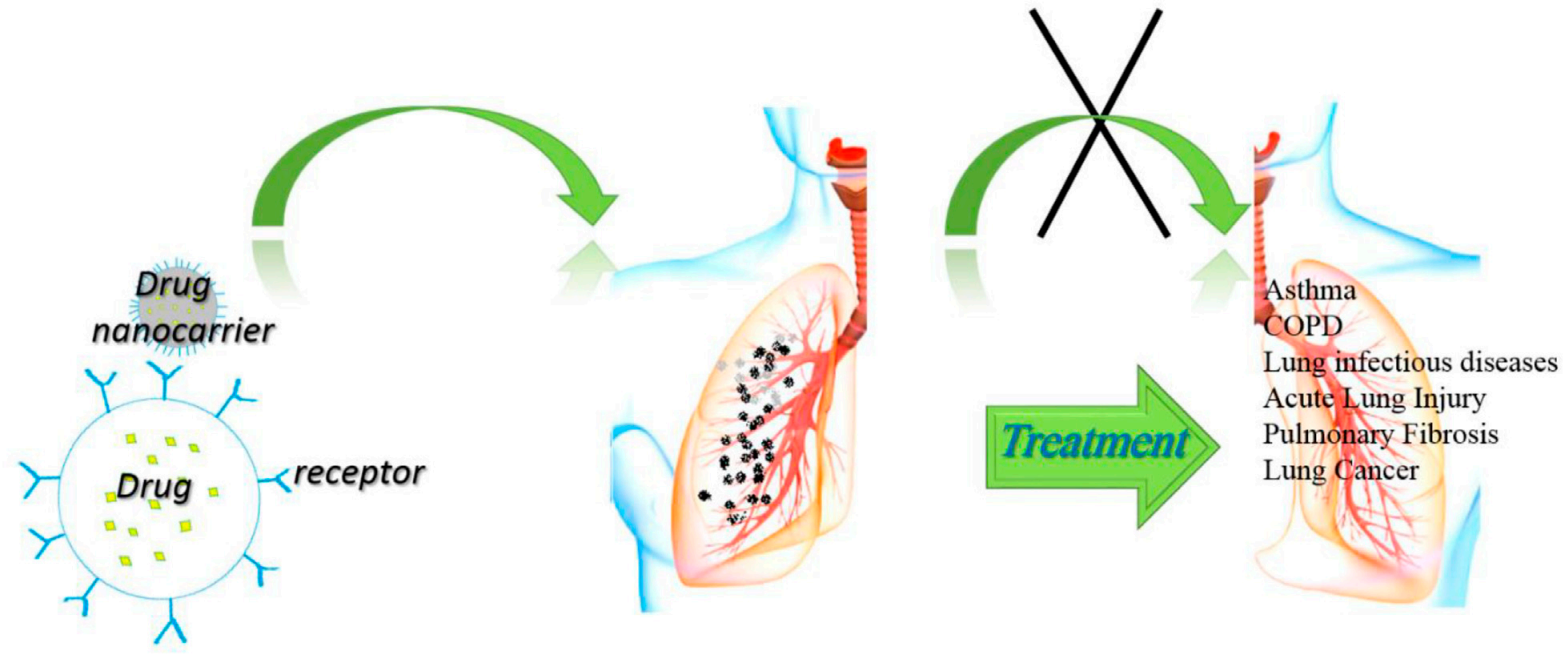
Summarized effects of nanoparticles on respiratory disorders.

This review article described the impact of different nanoparticles on different respiratory disorders. Nanoparticles showed therapeutic effects on respiratory disorders through various mechanisms such as anti-inflammatory, antioxidant, immunomodulatory, cell viability, proliferation, apoptosis, and necrosis mechanisms. Figure 9 summarizes the effects of nanoparticles on respiratory disorders.

### 5.1 Possible mechanisms of the effects of NPs on lung disorders

#### 5.1.1 Anti-inflammatory, antioxidant, and immunomodulatory effects

The size and structural properties of nanoparticles are among the most important components that can affect lung diseases. Various experiments on the effects of NPs on lung disorders, such as asthma, COPD, and other lung diseases, indicated that nanoparticles suppressed proinflammatory mediators, such as IL-6, IL-8, IL-1β, and TNF-α. In addition, the deactivation of CD4 and CD8 T cells in lung tissue and the reduction of their capability to generate proinflammatory cytokines was reported *in vivo* for lung disorder therapy using NPs (Das et al., 2022).

Lung cancer treatment with NPs could be associated with induced ROS, leading to apoptosis and necrosis (Nasrin et al., 2022). Nanoparticles can also downregulate the expression of Toll-like receptor 4, which is known as carcinogenesis in lung cancer cells and affects lung cancer treatment (Wang et al., 2022). In addition, it was indicated that treatment of lung cancer cells with NPs downregulated VEGF, HIF-1α, IL-8, and Bcl-2 genes but increased the gene expression of p53 (Huang et al., 2016b). Chlorin e6 loaded by iron oxide NPs linked with glucose significantly enhanced the uptake of Ce6 by lung cancer cells and produced more ROS, damaged the DNA of lung cancer cells, and thereby activated STING and upregulated the expression of IFN-β, HMGB1, and HSP90, indicating augmented immunogenicity of lung cancer cells (Yu et al., 2022). Silver nanoparticles (AgNP-DTa) prepared using an aqueous seed extract of *Derris trifoliata* showed antioxidant properties and anti-proliferative activities against A549 lung cancer cell lines (Cyril et al., 2019).

#### 5.1.2 Effect on cell viability and proliferation

The reduction effects of Rutin-loaded liquid crystalline nanoparticles (Rutin-LCNs) on NSCLC proliferation and migration were shown (Paudel et al., 2021), which is suggested to result from its inhibitory effect on the MMP-9 expression and prevent the degradation of the extracellular matrix (ECM) component. The combination of cell growth inhibition, cell cycle arrest, suppression of oncoproteins, and impeded cell migration due to HuR-FNP was also reported (Muralidharan et al., 2016). Biosynthesized zinc oxide NPs using the neem tree (100, 150, 200, and 250 μg/ml for 48 h) also significantly reduced cell (A549) viability (Rani et al., 2022). Antiproliferative and cytotoxic effect potentials of chitin and chitosan encapsulated gold nanoparticles (AuNPs) against human lung cancer cell line A549 were demonstrated (Panneer et al., 2022). The green synthesis of AgNPs using *Streptomyces hirsutus* also decreased the cell viability of A549 via ROS production (Pallavi et al., 2022). The cold atmospheric plasma (CAP) technique combined with iron oxide-based magnetic nanoparticles downregulated epidermal growth factor receptor (EGFR) and inhibited lung cancer cells by depressing pERK and pAKT (Li et al., 2019). Silver nanoparticles (AgNP-DTa) prepared using an aqueous seed extract of *D. trifoliata* showed antioxidant and anti-proliferative activities against A549 lung cancer cell lines (Cyril et al., 2019). Melatonin (MLT) and palladium nanoparticles (PdNPs) induced cytotoxicity through leakage of lactate dehydrogenase, increased intracellular protease, and reduced membrane integrity (Gurunathan et al., 2020).

Combining PdNPs with MLT elevated the levels of mitochondrial dysfunction by decreasing mitochondrial membrane potential (MMP), ATP content, mitochondrial number, and expression of the main regulators of mitochondrial biogenesis (Gurunathan et al., 2020). The biosynthesized AgNPs using the bark extract of *Toxicodendron vernicifluum* Tv-AgNPs selectively targeted and damaged the cancer cells through ROS generation in human lung carcinoma A549 cells (Saravanakumar et al., 2019). The effects of zinc oxide nanoparticles synthesized from *Euphorbia fischeriana* root (EF-ZnONPs) revealed cytotoxicity activity, inhibited the migration capability of A549 cells, diminished expression of Bcl-2, and enhanced expressions of Bid and Bax (Zhang H. et al., 2020). Cetuximab and doxorubicin-loaded dextran-coated Fe_3_O_4_ magnetic NPs suppressed cell proliferation of A549 (Zhang et al., 2019). TiO_2_ NPs also inhibited A549 cell proliferation and induced DNA damage and apoptosis via the activation of the intrinsic mitochondrial pathway (Wang Y. et al., 2015). Betulinic acid NPs on lung cancer cells (HKULC2, H1299, and H23) inhibited proliferation and metastatic ability and arrested the cell cycle through the downregulation of ATP-binding cassette and transporter G1 (ABCG1) oncogene expression (Zhao et al., 2020). Internalized fibroblast growth factor (FGF-2)-loaded NPs increased nuclear ERK1/2 content and resulted in lung cancer cell death (Miao et al., 2020). Amodiaquine (AQ)-loaded NPs (AQ NP) inhibited cell migration, reduced colony growth in A549 cells, inhibited AQ autophagy (increased LC3B-II levels), and improved apoptosis induction (caspase-3 levels) (Parvathaneni et al., 2020). Furthermore, treatment with SeNP-apigenin increased ROS production and oxidative stress in MCF-7 cells (Al-Otaibi et al., 2022). Selenium (Se) NPs also induced ROS-mediated necroptosis in PC-3 cells through TNF activation (Sonkusre and Cameotra, 2017). Generally, AgNP cytotoxicity depends on the dose, time, temperature, surface coatings, size, and cell type (Mousavi-Kouhi et al., 2021a). AgNP exposure reduced antioxidant enzymes, such as glutathione (GSH), elevated intracellular ROS levels and expression of ROS-responsive genes, and lipid peroxidation, leading to DNA damage, necrosis, and apoptosis (Tran and Le, 2013).

#### 5.1.3 Effect on apoptosis and necrosis

Tumor cells are characterized by their ability to evade apoptosis, and apoptosis could be used as an indicator for targeted cell treatment in cancer therapy (Akbarzadeh et al., 2022). Cationic poly-l-lysine-assisted magnetic iron oxide nanoparticles (PLL-MNP) in human lung cancer cells resulted in nuclear fragmentation and chromatin condensation (Wang X. et al., 2015). The selenium nanoparticles green-synthesized by apigenin (SeNP-apigenin) treatment in MCF-7 cells induced apoptosis, demonstrating that SeNP-apigenin could directly target Bcl-2, Bax, and caspase-3 and result in the discharge of cytochrome C from mitochondria into the cytosol, accompanied by the initiation of cell death, leading to permanent DNA damage and MCF-7 cell killing. Necrosis as an essential mechanism of cell death was induced by gold NPs in lung cancer cells with a low level of intracellular GSH (Liu et al., 2013). Another mechanism involved in necrosis induced by NPs is the induction of endothelial cell (EC) dysfunction through the release of VWF (von Willebrand factor). Dysfunction of EC induced by NPs can also result from the formation of ROS, inflammatory cytokines including IL-6 and IL-8, and/or the activation of the system of coagulation (Bauer et al., 2011). It was revealed that AgNPs reduced the apoptosis induced by TNF-α (Fehaid and Taniguchi, 2018). Curcumin nanoparticles (Cur-NPs) also induced apoptosis and caused G2/M arrest in both A549 and Calu-3 cell lines (Lee et al., 2016). Bevacizumab (Avastin, AV) nano-treatments also significantly suppressed A549 cell viability and activated apoptosis by NO level elevation (Abd-Rabou and Ahmed, 2019).

### 5.2 The dosage of NPs affecting respiratory disorders

The doses and duration of different types of NPs used on their effects on respiratory disorders were described in different sections of the review as summarized in Table 4.

**TABLE 4:** Doses and durations used for examining the effects of various nanoparticles on lung disorders.

Nanoparticle	Dose	Duration
AgNPs: silver nanoparticles	8–128 μg/ml	1, 6, 24 h
AuNPs: gold nanoparticles	20, 100 μg/ml–5.00 mg/ml	24, 48, 72 h
Al_2_O_3_ NPs	0.4, 2 mg/ml	24 h
Carbon nanotubes	0.4–20 μg/ml	24 h
CdO NPs: cadmium oxide nanoparticles	1 μg/ml	24 h
CdSe: cadmium selenide	330 μl	24 h
CS: chitosan	5 μg/m–2.5 mg/ml	6 and 24 h
LCN: liquid crystalline NPs	10–50 μg/ml	12, 24, and 48 h
LPB: liposome-in-bacteria	0.62–5 μmol/L	24 h
NiO NPs: nickel oxide NPs	5 μg/ml–0.24 mg/kg	6 weeks
ONP-302-NPs	1 mg	
QDs: quantum dots	5–20 μg/ml and 6 μg/kg	24 h
SBS: salbutamol sulfate	200 μg aerosolized	14 h
siRNA: small interfering RNA	5 μg–2.5 mg/kg	24 h and 3 days
SLN: solid lipid nanoparticles	30 µg/25 g/kg	1, 2, 3, and 6 days
TAC: tacrolimus	60 μg/mouse	
ZnO: zinc oxide nanoparticle	10, 20, and 40 μg/ml	

References were provided in the main text.

### 5.3 Future trends and prospects

The current review article mainly focuses on the effects of NPs on respiratory disorders based on *in vitro* and *in vivo* studies. However, to use different types of NPs in the treatment of various respiratory disorders, further studies, including *in vivo* and mainly clinical trials, should be performed on the following topics:

- Evaluation of the effect of different types of NPs more precisely.
- Accurately determining the duration of the effect of various types of nanoparticles.
- Find out the more effective doses of different types of NPs in particular.
- Examination of the different routes of administration of NPs, especially the effect of inhaler NPs.
- Assessment of the clinical outcome of different types of NPs in particular.

### 5.4 Conclusion

In this review article, the effects of different types of NPs on various lung disorders, including drug delivery, efficacy, and drug safety, based on the *in vitro* and *in vivo* findings and limited clinical trials were described. In asthma and COPD, different types of NPs reduced inflammatory cells and markers and improved lung oxidative stress and lung pathological changes in *in vitro* and *in vivo* studies. In PF also, although some studies indicated the therapeutic effects of NP drugs, other studies showed the induction of PF by NPS. Several *in vitro* and *in vivo* studies showed the effects of nanoparticle-based medicine as a promising tool for the treatment of lung infections by local antibiotic properties with low doses and reduced side effects. The effects of NPs on lung cancers (both primary and metastatic) were shown by increased drug delivery to selective lung cancer and lung metastases locations, as well as by increasing efficacy on lung cancer cells. The studies also showed that NP-based drugs are more effective than ordinary drugs. Some clinical studies also showed promising effects of NPs on the treatment of lung cancer. Various possible mechanisms of NPs on lung disorders were also summarized. However, more experimental and clinical studies are needed to evaluate the effects of nanomedicine on respiratory disorders and perform a meta-analysis to systematically assess the results of previous research before the production of nanoparticle-based medicine could be applied for clinical use. In addition, public knowledge regarding the effects of NPs and their benefits on various lung disorders should be encouraged.

## Figures and Tables

**Table:** 

**ABCG1**	ATP-binding cassette and transporter G1
**AG**	Andrographolide
**AgNPs**	Silver nanoparticles
**AHR**	Airways responsiveness
**AQ**	Amodiaquine
**AuNPs**	Gold nanoparticles
**BALF**	Bronchoalveolar lavage fluid
**Bcl-2**	B-cell lymphoma 2
**BDQ**	Bedaquiline
**BRNPS**	Bilirubin-based NPs
**CAP**	Cold atmospheric plasma
**CDDP**	Cisplatin
**CdO NPs**	Cadmium oxide nanoparticles
**CdSe**	Cadmium selenide
**COPD**	Chronic obstructive pulmonary disease
**CS**	Chitosan
**CuONPs**	Copper oxide nanoparticles
**Cur-NPs**	Curcumin-nanoparticles
**CXCL1**	Chemokine (C-X-C motif) ligand 1
**DOX**	Doxorubicin
**ECM**	Extracellular matrix
**EGFR**	Epidermal growth factor receptor
**FA**	Ferulic acid
**FACS**	Fluorescence-activated cell sorting
**GEB**	Gefitinib
**GSH**	Glutathione
**HA**	Hyaluronic acid
**HIF-1α**	Hypoxia-inducible factor 1
**IFN-γ**	Interferon-γ
**IL-1β**	Interleukin-1β
**IL-6**	Interleukin-6
**IL-8**	Interleukin-8
**INF-γ**	Interferon-gamma
**KC**	Chemokine
**LCN**	Liquid crystalline NPs
**LPB**	Liposome-in-bacteria
**LPS**	Lipopolysaccharide
**MCP-1**	Monocyte chemoattractant protein-1
**MERS-CoV**	Middle East respiratory syndrome coronavirus
**MMP**	Mitochondrial membrane potential
**MMP-9**	Matrix metalloproteinase-9
**MPO**	Myeloperoxidase
**NF-κβ**	Nuclear factor kappa B
**NiO NPs**	Nickel oxide nanoparticles
**NPs**	Nanoparticles
**OCR**	Rate of oxygen consumption
**PAMAM**	Poly(amidoamine) dendrimer
**PdNPs**	Palladium nanoparticles
**PEITC**	Phenethyl isothiocyanate
**PF**	Pulmonary fibrosis
**PG**	Gingerol
**QDs**	Quantum dots
**rCmP**	recombinant *Caryota mitis* profilin
**SARS-CoV**	Severe acute respiratory syndrome coronavirus
**SBS**	Salbutamol sulfate
**Si/sio**_**2**_	Silicon dioxide
**SiNPs**	Silica nanoparticles
**siRNA**	Small-interfering RNA
**SLIT**	Sublingual
**SLN**	solid lipid nanoparticles
**SM-LCN**	Surface-modified liquid crystalline NPs
**SPIONS**	Superparamagnetic iron oxide nanoparticles
**TAC**	Tacrolimus
**TLR4**	Toll-like receptor 4
**TNF-α**	Tumor necrosis factor-alpha
**Tob**	Tobramycin
**TQ**	Thymoquinone
**UCB**	Unconjugated bilirubin
**VEGF**	Vascular endothelial growth factor
**VIP**	Vasoactive intestinal peptide
**VWF**	von Willebrand factor
**Zns**	Zinc sulfide
**α-AN**	α-Alumina
